# Cannabis use and atherosclerotic cardiovascular disease: a Mendelian randomization study

**DOI:** 10.1186/s12872-023-03641-w

**Published:** 2023-12-13

**Authors:** Roxane de La Harpe, Tabea Schoeler, Christian W. Thorball, Aurélien Thomas, Zoltán Kutalik, Julien Vaucher

**Affiliations:** 1 Department of Medicine, Division of Internal Medicine, University Hospital of Lausanne, Rue du Bugnon 46, 1011 Lausanne, Switzerland; 2 Swiss Institute of Bioinformatics, 1015 Lausanne, Switzerland; 3 Department of Computational Biology, University of Lausanne, Lausanne, Switzerland; 4 Precision Medicine Unit, Biomedical Data Science Center, Lausanne University Hospital of Lausanne, Chemin des Roches 1a/1b, 1010 Lausanne, Switzerland; 5 Faculty Unit of Toxicology, CURML, Faculty of Biology and Medicine, University of Lausanne, Lausanne, Switzerland; 6 Centre universitaire de médecine et santé publique, Lausanne, Switzerland; 7 HFR Freiburg Kantonspital, Lausanne, Switzerland

**Keywords:** Cannabis, Atherosclerotic cardiovascular disease, Genetically predicted cannabis use, Causal inference, Mendelian randomization (MR), Modifiable risk factor

## Abstract

**Background:**

Association between cannabis use and development of atherosclerotic cardiovascular disease (ASCVD) is inconsistent and challenging to interpret, given existing study limitations.

**Methods:**

Sixty five independent single-nucleotide polymorphisms (SNPs), obtained from a genome-wide association study on lifetime cannabis use, were employed as genetic instruments to estimate the effects of genetically indexed cannabis use on risk of coronary artery disease (CAD) and acute ischemic stroke (IS) using a two-sample Mendelian randomization (MR) approach. Summary statistics on CAD (CARDIoGRAMplusC4D; 60,801 cases and 123,504 controls) and IS (MEGASTROKE; 34,217 cases and 406,111 controls) were obtained separately. A comprehensive review of the observational literature on cannabis use and CAD or IS was also performed and contrasted with MR results.

**Results:**

There was no causal effect of cannabis use on the risk of CAD (odds ratio (OR) per ever-users vs. never-users 0.93; 95% confidence interval (CI), 0.83 to 1.03) or IS (OR 1.05; 95%CI, 0.93 to 1.19). Sensitivity analyses yielded similar results, and no heterogeneity and directional pleiotropy was observed. Our meta-analysis of observational studies showed no significant association between ever use of cannabis with risk of CAD (k = 6 studies; OR_pooled_ = 1.23, 95%CI 0.78 to 1.69), nor with IS (k = 6 studies; OR_pooled_ = 1.22, 95%CI 0.95 to 1.50).

**Conclusion:**

Using a genetic approach approximating a clinical trial does not provide evidence consistent with a causal effect of genetic predisposition to cannabis use on CAD or IS development. Further studies are needed to replicate our findinds, an to investigate more precisely the risk of ASCVD in relation to the quantity, type, route of administration, or the age at exposure to cannabis.

**Supplementary Information:**

The online version contains supplementary material available at 10.1186/s12872-023-03641-w.

## Introduction

Cannabis is one of the most psychotropic substances used globally, with almost 4% of the population aged 15–64 years having consumed cannabis at least once in 2021 [1]. More evidence on the impact of cannabis use on health is thus necessary at population-wide and individual levels, especially with atherosclerosis cardiovascular diseases (ASCVD) accounting for 30% of globally deaths. While an association between cannabis use and risk of atherosclerosis cardiovascular diseases (ASCVD) has been reported numerous times [2–4], it remains inconclusive as to whether this link is causal in nature. In experimental studies, Cannabidiol (CBD) and tetrahydrocannabinol (THC), substances both present in cannabis, have been found to have potential beneficial effects against ASCVD development, mainly through anti-oxidative and anti-apoptotic effects [5–13]. Conversely, evidence also points towards adverse cardiovascular effects of THC, such as a decrease in myocardial contractility, vasospasm, tachycardia and systolic blood pressure increase, conditions that are known to promote ASCVD development [14–16].

Causality between cannabis use and ASCVD is challenging to assess in observational studies due to recall bias, inadequate exposure assessment, non-exhaustive inclusion of confounders or weak methodology design [17]. Since a deliberate exposure to cannabis would be unethical, a clinical trial, removing potential biases (e.g., confounding or reverse causation) in the cannabis-ASCVD association, is not possible, although representing the optimal method to test a clinical hypothesis. A genetic approach, mimicking a randomized trial, thus represents an asset to infer a causal association between a potential harmful exposure (cannabis) and a disease outcome (ASCVD) [18]. Recently, Zhao et al., using Mendelian randomization (MR) principles, did not find a causal association between cannabis use and ASCVD, but showed some evidence for a causal effect of cannabis use on small vessel stroke and atrial fibrillation [19]. However, their MR analysis was based on 10 genetic instruments only, since it was not derived from the largest and most recent genome-wide association study (GWAS) of cannabis use, which may have reduced the statistical power of their analysis.

To obtain more reliable results, here we used 65 independent genetic markers (from the most recent GWAS of cannabis use) to perform Mendelian randomization analyses. We included several sensitivity analyses and tested for the presence of pleiotropic effects of the instruments to ensure robust causal association results between cannabis consumption and both coronary artery disease (CAD) and ischemic stroke (IS). Further, we assessed whether adjusting for genetically indexed tobacco use altered the association. Finally, we performed a meta-analysis of published observational studies and contrasted the result with the causal estimates.

## Methods

### Principles of two-sample Mendelian randomization

Mendelian randomization (MR) is a statistical method using measured variation in single-nucleotide polymorphisms (SNPs) associated with an exposure to examine the causal effect of this exposure (cannabis use) on a disease outcome (CAD or IS). SNPs, used as genetic instruments, have to meet three assumptions to be valid instruments: i. relevance assumption (genetic instruments have to be robustly associated with the exposure of interest); ii. independence assumption (they should not be associated with any confounder of the exposure-outcome relationship); iii. Exclusion restriction assumption (they can affect the outcome only through the exposure) [18, 20].

Two-sample MR refers to the application of MR to summary genetic statistics estimated in two non-overlapping sets of individuals. The “first” sample is used for computing a genetic instrument for the exposure. The “second” sample is employed to estimate the instrument-outcome association. These two associations are then used to estimate the underlying causal effect [20].

### Genetic markers associated with ever use of cannabis

We used a publicly available GWAS computed from three distinct sources (ICC study, UK-Biobank and 23andMe), with a combined sample size of 184,765 participants of European ancestry, on ever use of cannabis (including 53,179 cases, 131,586 controls) (see Additional file 2, Supplementary Table 1 for details about studies included) [21]. All alleles in the GWAS were reported from the positive strand. Pasman et al. executed linkage disequilibrium clumping to eliminate genetically correlated SNPs (R^2^ < 0.001) and proposed 69 independent SNPs linked to ever use of cannabis, explaining 1.12% of the variance in cannabis use. Among these, we excluded four SNPs (rs11749751, rs2335349, rs3740390 and rs61942416) with discordant direction of effect among the three sources. We confirmed the independence of the SNPs with the LDpair tool (National Institutes of Health, LDlink, US https://ldlink.nih.gov/?tab=ldpair; as accessed on 2023, 4 Nov) (see Additional file 2, Supplementary Table 4). We selected then 64 SNPs; 5 which surpassed the conventional genome-wide significance threshold for genome-wide association with lifetime cannabis use (*p*-value < 5 × 10^−8^) and 59 other SNPs that passed a more lenient significance threshold (p-value < 5 × 10^−5^), but could be considered as an additional instrumental variable for the MR analysis (see Additional file 2, Supplementary Table 2) [22].

### Genetic markers associated with ASCVD

No publicly available GWAS repository on ASCVD was found. Therefore, we assessed the instrument-outcome association separately for CAD, using the Coronary Artery Disease Genome-wide Replication and Meta-analysis plus the Coronary Artery Disease 1000 Genomes-based GWAS (CARDIoGRAMplusC4 [23]) and IS, using the Multiancestry GWAS with stroke and stroke sub-types (MEGASTROKE [24]).

CARDIoGRAMplusC4D Consortium (www.cardiogramplusc4d.org/) involved 60,801 cases and 123,504 controls from 39 studies in a GWAS meta-analysis of CAD with 77% of European ancestry and about 70% of acute myocardial infarction (AMI). MEGASTROKE (www.megastroke.org) included 34,217 European cases and 406,111 European controls from 16 studies in a GWAS of IS.

The 64 SNPs associated with ever use of cannabis were matched and harmonized (i.e. matching the reference alleles) across the data sets. One SNP (rs80144387) was not available in CARDIoGRAMplusC4 data and was thus excluded from the analyses (see Additional file 2, Supplementary Table 2).

There was no overlap between participants from the GWAS of cannabis use and MEGASTROKE. Only two studies, including 3735 participants, contributed in both GWAS of cannabis use and CARDIoGRAMplusC4D (see Additional file 2, Supplementary Table 1 and Additional file 3, Supplementary Fig. 1).

### Observational association between ever use of cannabis and ASCVD

There was no meta-analysis in the literature reporting pooled observational association (correlation) estimates between cannabis use and ASCVD or, separately, for CAD or IS. We, therefore, conducted a random-effect meta-analysis, including studies assessing the association between cannabis use and ASCVD. Among the studies identified in a comprehensive literature search, we selected only prospective and retrospective observational studies. In addition, we only used studies that reported ever use of cannabis (compared with never users) as an exposure and a corresponding risk estimate (expressed as odds ratio (OR) or hazard ratio (HR)) for ASCVD, CAD or IS. Supplementary Fig. 2 in Additional file 3 presents the literature search strategy and Supplementary Tables 3 and 4 in Additional file 2 summarize the main characteristics of included and excluded studies, respectively.

### Statistical analysis

MR was conducted, using Stata v.17 (Stata, College Station, TX, USA, using mrobust package, available at: https://github.com/remlapmot/mrrobust) and R Statistical Software (v4.1.2; R Core Team 2021, using TwoSampleMR package v0.5.6, available at: https://mrcieu.github.io/TwoSampleMR/articles/index.html). Analyses were performed for CAD and IS separately.

We first generated a causal estimate for each instrumental variable (i.e., SNPs) by dividing the association of each SNP with risk of CAD/IS by the corresponding association with risk of ever use cannabis. The standard error (s.e.) was estimated using the delta method [25]. We then pooled together the individual causal effect estimates using fixed-effects (inverse variance weighted [IVW]) meta-analysis. As a sensitivity analysis, we also pooled together estimates using random-effects meta-analysis. To compare the pooled causal estimates to the pooled observational estimates, we transformed the summary estimates from meta-analysis into “users vs. non-users” of cannabis, as opposed to a per-1-log-unit increase in ever use of cannabis. To perform this transformation, we used estimates for the risk of CAD/IS in the general population and the prevalence of CAD/IS among never users of cannabis, as previously described [26]. A full description of the methodology is available in the Supplementary Methods. We also conducted a Steiger filtering analysis to test the direction of the causal estimate. This approach assumes that a valid instrumental variable should explain more variance for the exposure than for the outcome and identifies SNPs that do not satisfy this criterion (SNP-outcome correlation greater than the SNP-exposure correlation) [27]

### Strength of genetic instruments and power to detect a causal effect

We estimated instrument strength by calculating the proportion of variance in ever use of cannabis explained by each SNP. We used then F-statistic for each SNP individually and cumulatively assuming that F-statistics > 20 represents an acceptable correlation [28]. In the present study, the cumulative F-statistic was 29.1 minimizing the risk of weak instrument bias. Full details are provided in Additional file 1 and Additional file 2, Supplementary Table 2.

The power of our MR analysis to detect the same magnitude of association reported in the observational studies, using a two-sided α of 0.05, was 98% for both CAD and IS (see Additional file 2, Supplementary Table 5).

### Assessment of horizontal pleiotropy

To test the robustness of the causal estimation, we tested for the presence of pleiotropy. Egger Mendelian randomization (MR-Egger) method detects and corrects for the bias due to directional pleiotropy, allowing one or more SNPs to have pleiotropic effects, as long as the size of these pleiotropic effects is independent of the size of the SNPs effects on the exposure [29]. The methodology resembles conventional MR analysis (IVW), except that the intercept of the weighted linear regression is unconstrained (opposite as constrained equal to zero in IVW method) [29]. A low *p*-value for the MR-Egger intercept test suggests pleiotropy. The s.e. was obtained by bootstrap resampling 10′000 times. Finally, the *I*^*2*^ statistic in the context of MR-Egger quantifies weak instrument bias and was low in our analysis (*I*^*2*^ = 17% for CAD; and *I*^*2*^ = 39% for IS).

We then applied simulation extrapolation (MR-Egger-SIMEX implemented in Stata using the mrrobust package) to adjust the MR-Egger causal estimates to account for a potential NOME violation (NO Measurement Error assumption, the assumption that the SNP-exposure association is true) [30].

We also conducted a weighted median MR analysis (implemented in Stata using the mrobust package), which gives more weight to SNPs with homogeneous causal estimates (that is, close to the median causal estimate) even when up to 50% of the weight in the analysis arises from invalid SNPs [31]

### Sensitivity analysis

Tobacco consumption is a risk factor for ASCVD and shares a strong genetic correlation with use of cannabis [27, 28]. We then conducted a multivariable analysis adjusting for SNP-tobacco, to account for shared pathways with and/or potential confounding by tobacco, using summary statistics for the association of each of the 64 cannabis-related SNPs with tobacco. Smoking status was derived from 1,232,091 European individuals with 557,337 ever smoker phenotypes (vs never smoker) in the GWAS & Sequencing Consortium of Alcohol and Nicotine (GSCAN Consortium, https://conservancy.umn.edu/handle/11299/201564 downloads) [32]. Multivariable MR was conducted by regressing the SNP–cannabis estimates on SNP–CAD or -IS estimates adjusting for SNP–tobacco estimates. The s.e. was obtained by bootstrap resampling 10,000 times. Eight of 64 cannabis-related SNPs were not available in the GSCAN Consortium and therefore excluded from this analysis.

We finally computed three sensitivity analyses to test the coherence of our results. First, we restricted the level of genome-wide significance by the selection of genetic variants with a *p*-value< 5 × 10^−8^ (see Additional file 2, Supplementary Table 2). Second, as it was not possible to verify that the alleles reported by CARDIoGRAMplusC4D or MEGASTROKE have been correctly orientated, we selected SNPs in low linkage-disequilibrium with other SNPs (r2 < 0.001) within a clumping distance of 10,000 kb and removed palindromic SNPs if the allele frequency was close to 50% [33]. Third, as “ever use of cannabis” phenotype and genetic instruments derived from it can suffer from a lack of specificity, we repeated the analysis using SNPs from a recent GWAS of cannabis use disorder, comprising 14,080 cases and 343,726 controls of unrelated individuals from European ancestry (Psychiatric Genomic Consortium, https://pgc.unc.edu/for-researchers/download-results/downloads, more details in Additional file 1) [34]

## Results

### Observational association between ever use of cannabis and risk of CAD and IS

**Fig. 1:**
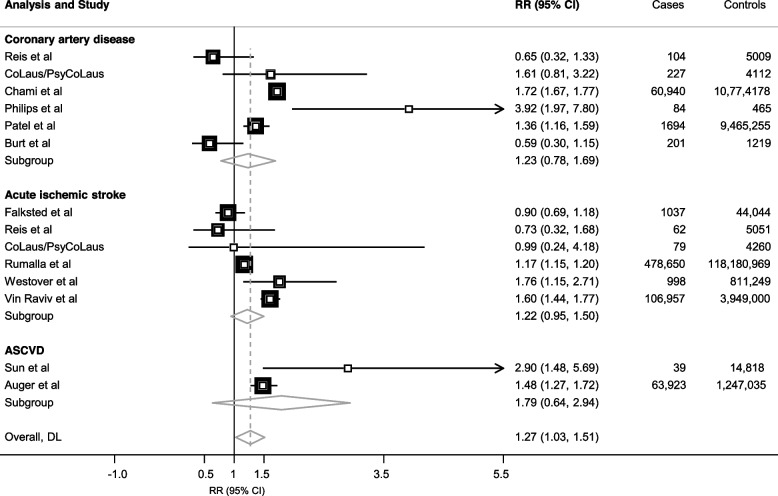
Meta-analysis of observational studies reporting association between cannabis and atherosclerotic cardiovascular disease. Meta-analysis uses a random-effects model, DerSimonian and Lair methods (DL). Studies are sorted by type of outcome (coronary artery disease, ischemic stroke or global ASCVD analysis). Relatives risk (RR) and 95% confidence intervals (CI) express the risk of ASCVD for “ever use of cannabis” (compared with never use). For additional information on each study, see Additional file 2, Supplementary Table 1. Supplementary Fig. 4 and 5 provide meta-analysis stratified by outcome and type of risk ratio

Twelve different studies met our primary research criteria, with results for several of our outcomes of interest for two of them (Reis et al. study [35] and CoLaus/PsyCoLaus et al. study [36]). Details of the outcomes of these studies can be found in Supplementary Table 3. It is worth noting the variability of the ASCVD outcomes, sometimes including only cardiovascular mortality in Sun et al. study [3] or a broader outcome such as cardiovascular hospitalizations including heart, pulmonary vascular, cerebrovascular disease and hypertension in Auger et al. study [37]. Six of them reported ever use of cannabis (compared with no use) along with CAD status and six others measured ever use of cannabis along with IS. When meta-analysing these estimates, ever use of cannabis was not significantly associated with risk of CAD using random-effects modelling (OR_pooled_ 1.23, 95% confidence interval (CI) 0.78 to 1.69, I^2^ 90.8%; see Additional file 3, Supplementary Fig. 3), nor with risk of IS (OR_pooled_ 1.22, 95% CI 0.95 to 1.50, I^2^ 85.4%; see Additional file 3, Supplementary Fig. 4). Combining studies observing CAD or IS development individually with the two studies assessing only aggregated ASCVD [3, 37] (encompassing 714,938 cases and 144 million controls), ever use of cannabis was significantly associated with overall ASCVD (OR_pooled_ 1.27, 95% CI 1.03 to 1.51, *p*-value< 0.001, I^2^ 97.3%), using a random-effects modelling (Fig. 1). The cannabis-ASCVD association was similar when only prospective studies assessing aggregated ASCVD as outcome were selected (see Additional file 3, Supplementary Fig. 5). Egger’s test via funnel plot asymmetry, as measured a linear regression of the effect estimates on their standard errors weighted by their inverse variance, was not significant (0.64, 95% CI − 0.54 to 1.81, *p*-value = 0.3), indicating an unlikely publication bias.

### Causal effect estimates of ever use of cannabis on risk of CAD and IS

**Fig. 2:**
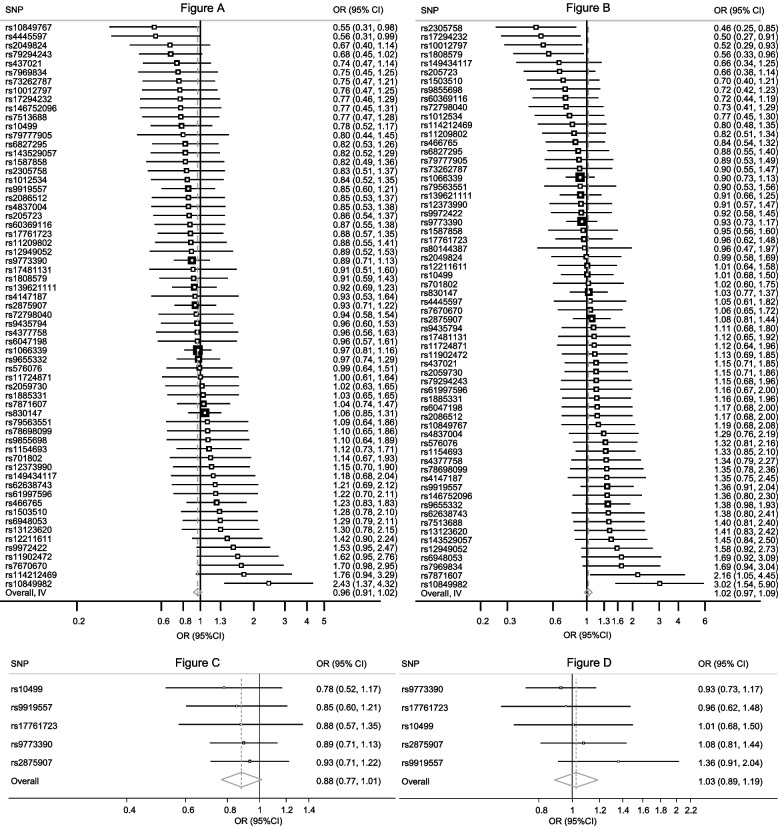
Effect of each cannabis use-associated SNP on risk of CAD (**A**) or IS (**B**) and when using a stringent threshold for SNPs’ selection for CAD (**C**) or IS (**D**). Meta-analysis of the association of genetically instrumented use of cannabis and risk of CAD for the 63 single-nucleotide polymorphisms (SNPs) (**A**) and of IS for the 64 SNPs (**B**). The second meta-analysis shows results when using 5 SNPs which surpassed the conventional genome-wide significance threshold for genome-wide association with lifetime cannabis use (*p*-value < 5 × 10^−8^) for CAD (**C**) and for IS (**D**). Odds ratios (OR) and 95% confidence intervals (CI) express the risk of event per-1-log unit increase in ever use of cannabis. Meta-analysis uses a fixed effect model

The 64 SNPs associated with ever use of cannabis explained 1% of its variance. In MR analysis, ever use of cannabis was not significantly causally associated with risk of CAD (OR per-1-log unit in ever use of cannabis [derived by fixed-effect meta-analysis of individual causal effects estimates of SNPs], 0.97, 95% CI 0.92 to 1.02, *p*-value = 0.19; Fig. 2.A and see Additional file 3, Supplementary Fig. 6). Similarly, no causal effect was found when IS was assessed as the outcome (OR per-1-log unit in ever use of cannabis, 1.03, 95% CI 0.98 to 1.09, *p*-value = 0.41; Fig. 2.B and see Additional file 3, Supplementary Fig. 7). Random-effects meta-analysis showed converging results (OR 0.97, 95% CI 0.92 to 1.02, p-value = 0.19 for CAD and 1.03, 95% CI 0.97 to 1.09, p-value = 0.38 for IS). Steiger filter showed correct causal direction for both CAD and IS overall (p-value< 0.001 for both) and for each SNP, separately.

**Fig. 3:**
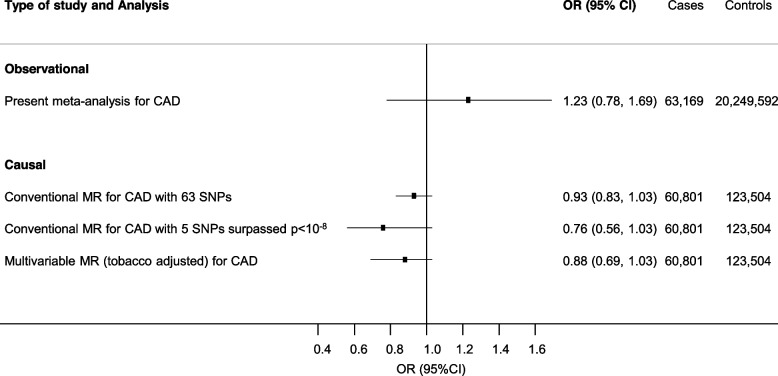
Comparison of observational and causal estimates for cannabis use and risk of CAD. Observational estimates are provided according to the meta-analysis reported in Fig. 1 restricted to coronary artery disease, as separate outcome for ever use of cannabis. Causal estimates represent population-based association derived by conventional (Fig. 2) and multivariable Mendelian randomization. The method to derive the population-based OR of ASCVD among users of cannabis compared with non-users is described in Additional file 1

**Fig. 4:**
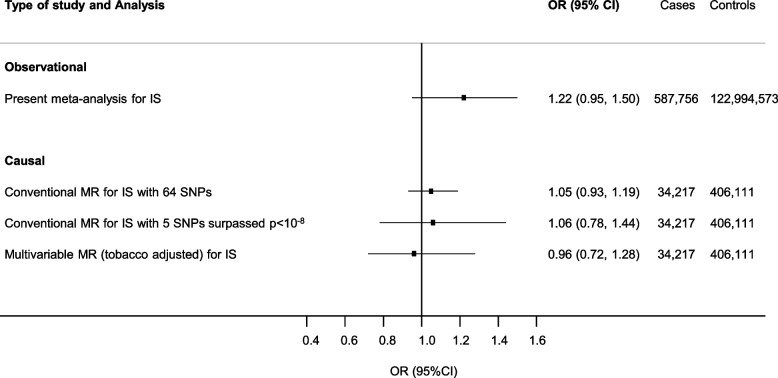
Comparison of observational and causal estimates for cannabis use and risk of IS. Observational estimates are provided according to the meta-analysis reported in Fig. 1 restricted to ischemic stroke, as separate outcome for ever use of cannabis. Causal estimates represent population-based association derived by conventional (Fig. 2) and multivariable Mendelian randomization. The method to derive the population-based OR of ASCVD among users of cannabis compared with non-users is described in Additional file 1

The MR estimate transformed in population-based OR (OR per users vs. non-users for CAD, 0.93, 95% CI 0.83 to 1.03; Fig. 3 and OR for IS, 1.05, 95% CI, 0.93 to 1.19; Fig. 4) was consistent with estimates derived from observational analysis for CAD (test for heterogeneity between group, *p*-value = 0.185) and IS (test for heterogeneity between group, *p*-value = 0.053).

### Assessment of pleiotropic effects of the genetic markers

We did not find evidence against the null hypothesis of no directional pleiotropy of the genetic markers using MR-Egger (*P*-value for pleiotropy of 0.766 for CAD and 0.653 for IS). The causal estimates derived from MR-Egger, MR-Egger adjusted for simulation extrapolation (SIMEX) and weighted median MR produced consistent causal estimate compared with conventional MR estimates for CAD (see Additional file 2, Supplementary Table 6 and Additional file 3, Supplementary Fig. 8) and IS (see Additional file 2, Supplementary Table 7 and Additional file 3, Supplementary Fig. 9).

### Sensitivity analysis

Adjusting for smoking in multivariable MR did not show evidence of shared pathways and/or confounding with a causal effect estimate of CAD and IS (OR per-1-log unit 0.94, 95% CI 0.84 to 1.05 for CAD; and 0.97, 95% CI 0.86 to 1.12 for IS, ORs per users vs. non-users are shown in Figs. 3 and 4). The pattern of the MR estimates did not change after using a more stringent threshold for the selection of genetic variants (*p*-value < 5 × 10^−8^) (Figs. 2, 3 and 4 and see Additional file 2, Supplementary Table 8). The effect was consistent with a more conservative approach selecting genetic instruments (removal of palindromic SNP with intermediate minor allele frequency) (see Additional file 2, Supplementary Table 9), or when we estimated the causal effect for cannabis use disorder as exposure (see Additional file 2, Supplementary Table 10).

## Discussion

Using a genetically informed causal inference approach, this study provides no evidence that genetically indexed cannabis use has a causal effect on CAD or IS risk, a result robust to a range of sensitivity analyses. This finding is in line with previous MR results relying on less powerful genetic instruments [19]. They are also consistent with a meta-analysis which did not find serious cardiovascular events in a randomized controlled trial (RCT) using medical cannabinoids [38]. These findings are important from individual- and public health perspectives, considering the increase in prevalence of medical and recreational use of cannabis [1]

Our meta-analysis, combining estimates obtained in observational studies, showed no significant. association between cannabis use and CAD or IS development individually, which is coherent with our MR findings. However, we found a borderline significant association between cannabis use and overall ASCVD development when we combined the observational studies assessing cannabis and CAD and IS individually with observational studies assessing cannabis and mortality or incident ASCVD. This result was consistent when analyzing only prospective studies with ASCVD or ASCVD mortality. Whereas these results were not affected by publication bias, there was substantial levels of heterogeneity between studies, which can lead to this spurious significant association between cannabis use and overall ASCVD development. This highlights the difficulty to interpret the association of a behavior on health using observational studies. These observational studies might suffer from confounding factors and competing risk after exposure. Moreover, comparison between different length of follow-up and different case definition could potentially lead to biased results**.** Finally, larger samples in meta-analyses receiving more weight, the pooled effect would largely reflect the effects obtained from larger studies (i.e no change in the pooled effect when Sun et al. study was removed see Additional file 3, Supplementary Fig. 10). It is noteworthy that Auger et al. study [37], driving the effect towards an association in the overall meta-analysis only included women.

Using Mendelian randomization limiting these biases, our study shows no evidence of association between cannabis use and either coronary artery disease or ischemic stroke, despite numerous observational studies reporting a detrimental association [2–4, 39–41]. Because a GWAS reporting ASCVD as outcome was not available, we were not able to compare with a genetic approach the evidence of an association between cannabis ever use and development of ASCVD, as found in our meta-analysis. Finally, differences in cannabis doses, formulations, exposition-time and pattern of use can have different and divergent effects and contributions on the occurrence of different ASCVDs and thus lead to confusion when testing their contribution together and for an overall ASCVD outcome. Indeed, cannabis use exposed to hundreds of cannabinoids, the effects of most of which are not known, especially with regard to their affinity to cannabinoid receptor (CBR). Even the two main cannabinoids, namely cannabidiol (CBD) and tetrahydrocannabinoid (THC), involved in the use of cannabis have different known or hypothesized effects of the two substances involved in the use of cannabis. The endocannabinoid system includes two receptors: cannabinoid receptor 1 (CBR1) and 2 (CBR2) with different biological roles [12]. THC is an agonist of the CBR1 receptor, which, via the autonomic nervous system, induces an increase in heart rate and blood pressure [12, 42], thus suggested as a trigger for ASCVD in case of predisposition [43]. Conversely, high-doses of THC, translate into a decrease in heart rate, as well as blood pressure with a decrease in cerebral flow, which is suggested as a mechanism for the development of IS [42]. Activation of CBR2 has been shown to regulate inflammation and may limit the production of oxidized lipoprotein by modulating the effect of CBR1 in the development of atherosclerosis. Whereas the mechanisms of action of CBD, particularly in relation to inflammation, remain obscure, indirect effects on anandamide which could modulate CBR1 and CBR2 have been considered for [5, 7, 11, 12, 42]. Therefore, further MR studies with SNPs specific to THC, CBD use or CBR1/CBR2 agonist or antagonist may distinguish the effects of the two main substances composing cannabis.

One limitation of our study is weak instrumental variables (that did not achieve GWAS significance) can lead to downward bias and hence loss in statistical power [44]. The availability of only a few SNPs reaching the conventional genetic significance threshold of < 5 × 10^–8,^ further reduces power. However, the F-statistic provided evidence against weak instrumental bias. Second, when exposure and outcome datasets are not overlapping, as in the present study, there exists the risk of underestimating the true causal effect estimate. However, sensitivity analyses are concordant with main findings and reinforce the confidence on our results. Other limitations include that our study did not allow the investigation of the risk of ASCVD in relation to the quantity, type, route of administration, or the age at exposure to cannabis. Moreover, capturing patterns of cannabis use in individuals presents inherent complexities attributable to multiple contributing factors, such as recall biases, respondents’ reticence, uncertainty regarding the composition of the substances and the utilization of survey instruments that may fall short in effectively delineating the diverse spectrum of users. Dichotomizing individuals into binary categories of ever versus never users, as is commonplace in various studies, might oversimplify the complexity of substance use behaviors and consequently its effect on health. And although the sensitivity analysis we conducted, using cannabis use disorder as an exposure to better account for chronicity and the amount of cannabis consumed, confirmed the absence of evidence of an effect, our results cannot be generalized to other groups of individuals exposed to cannabis (e.g., early onset users, users of high-potency cannabis) that may experience more severe health consequences. Larger GWAS studies are then necessary to circumvent the use of weak instruments and allow the identification of sub-phenotypes of exposure. Finally, all genetic summary statistics are from European ancestry, except for the CardioGRAMplusC4D (23% of participants from a different ethnical background). Differences in ancestry may mean, for example, that a genetic association with cannabis may be true in that specific population, but, due to differences in linkage disequilibrium, this will not be the case in a different ancestry group, which in turn can affect the causal association with ASCVD.

## Conclusion

Our genetic approach, approximating a randomized control trial that would be unethical in these circumstances, showed no evidence consistent with a causal effect of genetic liability to cannabis use on risk of CAD or IS. Knowing the burden of ASCVD and the frequency of cannabis use in the general population, further studies are needed to replicate our findings, and to investigate more precisely the risk of ASCVD in relation to the quantity, type, route of administration, or the age at exposure to cannabis.

### Supplementary Information

**Additional file 1.** Supplementary Methodology.

**Additional file 2.** Supplementary Tables.

**Additional file 3.** Supplementary Figures.

## Data Availability

The datasets analysed during the current study are available in the International Cannabis Consortium (ICC)] repository, https://www.ru.nl/bsi/research/group-pages/substance-use-addiction-food-saf/vm saf/genetics/international-cannabis-consortium-icc/ [21]; in the CARDIoGRAMplusC4D Consortium repository, http://www.cardiogramplusc4d.org/data-downloads/ [23]; in the MEGASTROKE Consortium repository, https://www.megastroke.org/ [24]; in the Repository for U of M, https://conservancy.umn.edu/handle/11299/201564 [33]; in the Psychiatric Genomic Consortium repository, https://www.med.unc.edu/pgc/download-results/ [34]. Codes and scripts are available upon reasonable request.
