# Supplementary material for: Cannabis use and atherosclerotic cardiovascular disease: a Mendelian randomization study
Source: BMC Cardiovasc Disord. 2023 Dec 13;23:611. doi: 10.1186/s12872-023-03641-w (PMC10717446; doi:10.1186/s12872-023-03641-w)
Supplement: Supplementary file 1 — Additional file 1. Supplementary Methodology. [file 12872_2023_3641_MOESM1_ESM.docx]

**Additional File 1** **- Supplementary Methodology**

**Transformation of MR causal estimates from a per-1-log unit increase in ever use of cannabis to users vs. non-users**

It is based on Ross et al’s equations[1] and descriptions below come from Vaucher et al[2]:

Given:

1. Prevalence of global lifetime cannabis use = 0.272 for European population.[3]
2. Prevalence of CAD and IS in non-users of cannabis from an observational cohort.
3. Odds ratio for CAD and IS associated with genetically determined cannabis use (users vs. non users) at the population level
4. Genetic association with CAD and IS as function of genetic association with use of cannabis (where causal genetic effects are expressed as Log OR per allele for both outcome and use of cannabis)

The following can be calculated:

1. Calculated population prevalence of CAD and IS: **(A × B × C) + (1 - A) × B**
2. Estimated prevalence of CAD and IS in individuals with a theoretical increase in risk of use of cannabis of *e* = 2.72 fold: **(A × exp(1) × B × C) + (1 - A × exp(1)) × B**
3. Estimated odds ratio for CAD and IS per *e* = 2.72 fold increase in risk of use of cannabis: **F/E**

It results that:

1. $\mathbf{exp}\left( \mathbf{D} \right)\mathbf{= G =}\frac{\boldsymbol{F}}{\boldsymbol{E}}\mathbf{=}\frac{\boldsymbol{(A \times exp(1) \times B \times C) + (1 - A \times exp(1)) \times B}}{\left( \boldsymbol{A \times B \times C} \right)\mathbf{+}\left( \mathbf{1 - A} \right)\boldsymbol{\times B}}$

As C is the only unknown variable, the association between genetically determined use of cannabis (cannabis users vs. non-users) and risk of ASCVD (expressed as an odds ratio) at the population level can be calculated using algebraic transformations and (H) can be simplified into:

1. $\mathbf{C}\mathbf{=}\mathbf{1}\mathbf{+}\frac{\mathbf{(}\mathbf{1}\mathbf{-}\exp\left( \mathbf{D} \right)\mathbf{)}}{\left( \mathbf{ex}\mathbf{p} \left( \mathbf{D} \right)\mathbf{-}\exp\left( \mathbf{1} \right) \right)\boldsymbol{\times}\mathbf{A)}}$

**Variance and F-statistic calculation for each SNP and cumulatively**

The proportion of variance (conceptually similar to the R^2^) in use of cannabis was computed for each SNP based on the formula provided by Shim et al.[4]

$$\boldsymbol{R}\mathbf{2}\mathbf{=}\frac{\begin{aligned} \text{2}\text{β}\text{2}\text{ }\text{x M}\text{AF}\text{ x }\text{(1}\text{ }\text{-}\text{ }\text{M}\text{AF}\text{)} \end{aligned}}{\text{2}\text{β}\text{2}\text{ x M}\text{AF}\text{ x }\left( \text{1}\text{ }\text{-}\text{ }\text{M}\text{AF} \right)\text{ +}\text{ }\left( \text{se}\left( \text{β} \right) \right)\text{2}\text{ }\text{x 2}\text{N}\text{ x }\text{M}\text{AF}\text{ x }\text{(1}\text{ }\text{-}\text{ }\text{M}\text{AF}\text{)}}$$

with β, effect size (β coefficient) for a given SNP; MAF, minor allele frequency; se(β) standard error of effect size and *N*, sample size.

Total proportion of variance explained for the association between the SNPs and the use of cannabis result in cumulative effect of each SNP.

The F-statistics was then calculated as follow, according to Pierce et al[5] :


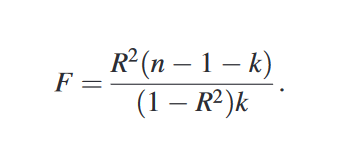


with, n, sample size; k, number of instrument variables used

**Association between cannabis use and ASCVD in CoLaus/PsyCoLaus cohort study**

The detailed description of the recruitment of the PsyCoLaus study and the follow-up procedures have been described previously[6]. Briefly, this cohort study is a population-based cohort exploring the biological, genetic and environmental determinants of ASCVD. A non-stratified, representative sample of the population of Lausanne (Switzerland) was recruited between 2003 and 2006 based on the following inclusion criteria: (1) age 35–75 years and (2) willingness to participate. The third follow-up occurred 15 years after the baseline survey.

ASCVD, comprised non-fatal acute myocardial infarction (AMI), symptomatic coronary artery disease with greater than 50% stenosis treated by percutaneous coronary intervention or coronary artery bypass graft (CHD), and fatal and non-fatal ischaemic stroke (including transient ischaemic attack) were comprehensively collected and independently adjudicated.

Participants were invited to attend the outpatient clinic at Lausanne University Hospital for a specific interview conducted by psychologists with questions relative to drug use, abuse and dependence, constructed the PsychoLaus cohort.

6’770 participants were involved at baseline and 5’128 took part to the PsyCoLaus Cohort. We excluded participant with missing data at baseline (n=434), previous ASCVD (n=93) and loss of follow-up (n=262).

Cox proportional hazard regressions with time of follow-up as the underlying time variable were used to test associations between cannabis use and incident ASCVD or stroke or coronary artery disease only. Co-variables were demographic variables (age and sex) and risk factors for ASCVD (body mass index, hypertension arterial and diabetes mellitus). Outcomes were censored if a participant was lost to follow-up, died from a non-cardiovascular cause, or if the end of available follow-up was reached.

**Genome-wide association study meta-analysis of cannabis use disorder**

We used summary-level data from the publicly available GWAS of Johnson et al, comprising 374’287 European participants, which explained phenotype defined as a lifetime diagnosis of cannabis abuse or dependence (according to DSM-IV or ICD_10). The GWAS was performed of 20 samples (18 from the Psychiatric Genomic Consortium Substance Use Disorder working group, on from iPSYCH and one from decoDE, More information about recruitment, measurement instruments and phrasing of the questions about lifetime cannabis use can be found in the supplementary material of Johnson et al.[7] We selected SNPs with p-value that were associated with cannabis use disorder (p-value<5x10^-5^) and in low linkage-disequilibrium with other SNPs (R^2^ < 0.001) within a clumping distance of 10,000 kb. Palindromic SNPs with intermediate minor allele frequency were removed because we cannot assumed that all alleles were correctly reported in the positive strand in cannabis use disorder GWAS. A more restricted threshold with p-value <5x10^-8^ was initially computed but only 2 SNPs remained, which was not sufficient for two-sample MR analysis (see Supplementary Table 10).

**References**

1. Ross S, Gerstein HC, Eikelboom J, Anand SS, Yusuf S, Paré G. Mendelian randomization analysis supports the causal role of dysglycaemia and diabetes in the risk of coronary artery disease. Eur Heart J. 2015 Jun 14;36(23):1454–62.

2. Vaucher HB Nadine Hausler, David Nanchen, Marie Méan, Pedro Marques Vidal, Julien. Comparison of Swiss and European risk algorithms for cardiovascular prevention in Switzerland - Hadrien Beuret, Nadine Hausler, David Nanchen, Marie Méan, Pedro Marques-Vidal, Julien Vaucher,. European Journal of Preventive Cardiology [Internet]. 2020 Feb 23 [cited 2020 Apr 7]; Available from: https://journals.sagepub.com/doi/full/10.1177/2047487320906305?rfr_dat=cr_pub%3Dpubmed&url_ver=Z39.88-2003&rfr_id=ori%3Arid%3Acrossref.org&journalCode=cprc

3. European Monitoring Centre for Drugs and Drug Addiction. European drug report 2021: trends and developments. [Internet]. LU: Publications Office; 2021 [cited 2022 Jul 4]. Available from: https://data.europa.eu/doi/10.2810/18539

4. Shim H, Chasman DI, Smith JD, Mora S, Ridker PM, Nickerson DA, Krauss RM, Stephens M. A multivariate genome-wide association analysis of 10 LDL subfractions, and their response to statin treatment, in 1868 Caucasians. PLoS One. 2015;10(4):e0120758.

5. Pierce BL, Burgess S. Efficient design for Mendelian randomization studies: subsample and 2-sample instrumental variable estimators. Am J Epidemiol. 2013 Oct 1;178(7):1177–84.

6. Firmann M, Mayor V, Vidal PM, Bochud M, Pécoud A, Hayoz D, Paccaud F, Preisig M, Song KS, Yuan X, Danoff TM, Stirnadel HA, Waterworth D, Mooser V, Waeber G, Vollenweider P. The CoLaus study: a population-based study to investigate the epidemiology and genetic determinants of cardiovascular risk factors and metabolic syndrome. BMC Cardiovasc Disord. 2008 Mar 17;8:6.

7. Johnson EC, Demontis D, Thorgeirsson TE, Walters RK, Polimanti R, Hatoum AS, Sanchez-Roige S, Paul SE, Wendt FR, Clarke TK, Lai D, Reginsson GW, Zhou H, He J, Baranger DAA, Gudbjartsson DF, Wedow R, Adkins DE, Adkins AE, Alexander J, Bacanu SA, Bigdeli TB, Boden J, Brown SA, Bucholz KK, Bybjerg-Grauholm J, Corley RP, Degenhardt L, Dick DM, Domingue BW, Fox L, Goate AM, Gordon SD, Hack LM, Hancock DB, Hartz SM, Hickie IB, Hougaard DM, Krauter K, Lind PA, McClintick JN, McQueen MB, Meyers JL, Montgomery GW, Mors O, Mortensen PB, Nordentoft M, Pearson JF, Peterson RE, Reynolds MD, Rice JP, Runarsdottir V, Saccone NL, Sherva R, Silberg JL, Tarter RE, Tyrfingsson T, Wall TL, Webb BT, Werge T, Wetherill L, Wright MJ, Zellers S, Adams MJ, Bierut LJ, Boardman JD, Copeland WE, Farrer LA, Foroud TM, Gillespie NA, Grucza RA, Harris KM, Heath AC, Hesselbrock V, Hewitt JK, Hopfer CJ, Horwood J, Iacono WG, Johnson EO, Kendler KS, Kennedy MA, Kranzler HR, Madden PAF, Maes HH, Maher BS, Martin NG, McGue M, McIntosh AM, Medland SE, Nelson EC, Porjesz B, Riley BP, Stallings MC, Vanyukov MM, Vrieze S, Davis LK, Bogdan R, Gelernter J, Edenberg HJ, Stefansson K, Børglum AD, Agrawal A. A large-scale genome-wide association study meta-analysis of cannabis use disorder. Lancet Psychiatry. 2020 Dec;7(12):1032–45.
