# Supplementary material for: Cannabis use and atherosclerotic cardiovascular disease: a Mendelian randomization study
Source: BMC Cardiovasc Disord. 2023 Dec 13;23:611. doi: 10.1186/s12872-023-03641-w (PMC10717446; doi:10.1186/s12872-023-03641-w)
Supplement: Supplementary file 2 — Additional file 2. Supplementary Tables. [file 12872_2023_3641_MOESM2_ESM.docx]

**Additional File 2** - **Supplementary Tables**

**Supplementary Table 1.** Studies included in the cannabis-GWAS used in our analysis

| Study | Country | *N total* | Reference/PMID |
| --- | --- | --- | --- |
| *International Cannabis Consortium* | | | |
| ALSPAC | UK | 2976 | 22507743 |
| BLTS | Australia | 721 | 23187020 |
| CADD | US | 853 | Not published |
| EGCUT1 | Estonia | 2765 | 15133739 |
| EGCUT2 | Estonia | 970 | 15133739 |
| FinnTwin | Finland | 1029 | 23298696 |
| HUVH | Spain | 981 | 25284319 |
| MCTFR | US | 6241 | 23363460 |
| NTR | Netherlands | 4653 | 20477721 |
| QIMR | Australia | 6778 | 17988414 |
| TRAILS | Netherlands | 1226 | 18763693 |
| Utrecht | Netherlands | 1173 | 20925969 |
| Radar | Dutch | 338 | 25466800 |
| SYS | Canada | 551 | 25454417 |
| TwinsUK | UK | 2078 | twinsuk.ac.uk |
| Yale Penn African American | US | 2660 | 24166409 |
| Yale Penn European American | US | 1964 | 24166409 |
|  | | | |
| *23andMe* | | | |
| 23andMe | - | 22683 | - |
|  | | | |
| *UK-Biobank* | | | |
| UK-Biobank | UK | 126785 | 25826379 |

Overview of studies included in the GWAS from Pasman et al[1]. In orange, studies which were also found in CARDIoGRAMplusC4D GWAS[2]. No similar studies were found in MEGASTROKE[3].

**Supplementary Table 2.** Summary of the 65 SNPs associated with use of cannabis (by decreasing p-value)

| SNP | EA | EAF | Beta | Standard error | p-value | Chromosome | Sample size | R^2^ | F-Statistic |
| --- | --- | --- | --- | --- | --- | --- | --- | --- | --- |
| rs2875907 | A | .3524 | .0712 | .0086 | 9.381e-17 | 3 | 184765 | .0003708 | 68.51757 |
| rs9919557 | C | .386 | .0549 | .0085 | 9.935e-11 | 11 | 184765 | .0002257 | 41.70699 |
| rs10499 | A | .6513 | .0532 | .0087 | 1.134e-09 | 16 | 184765 | .0002023 | 37.38496 |
| rs9773390 | C | .0673 | .1714 | .0294 | 5.659e-09 | 8 | 57980 | .0005859 | 33.96819 |
| rs17761723 | T | .3462 | .0473 | .0085 | 3.236e-08 | 17 | 184765 | .0001676 | 30.96076 |
| rs466765 | A | .2092 | .0565 | .0104 | 5.884e-08 | 6 | 184765 | .0001597 | 29.50943 |
| rs1154693 | G | .8538 | .063 | .0117 | 6.921e-08 | 3 | 184765 | .0001569 | 28.98953 |
| rs12373990 | T | .1171 | .0667 | .0128 | 1.989e-07 | 22 | 184765 | .0001469 | 27.14988 |
| rs6827295 | G | .7139 | .0467 | .0091 | 2.490e-07 | 4 | 184765 | .0001425 | 26.33232 |
| rs12211611 | G | .8084 | .0538 | .0104 | 2.552e-07 | 6 | 184765 | .0001448 | 26.75685 |
| rs1066339 | A | .1683 | .1472 | .0286 | 2.719e-07 | 6 | 57980 | .0004567 | 26.478 |
| rs9972422 | G | .2907 | .0462 | .009 | 2.754e-07 | 15 | 184765 | .0001426 | 26.34735 |
| rs437021 | C | .5408 | .0422 | .0082 | 2.770e-07 | 1 | 184765 | .0001433 | 26.48103 |
| rs576076 | A | .2534 | .0477 | .0094 | 4.431e-07 | 11 | 184765 | .0001393 | 25.74664 |
| rs114212469 | T | .0207 | .1578 | .0317 | 6.230e-07 | 4 | 149468 | .0001658 | 24.77556 |
| rs1808579 | T | .4792 | .0408 | .0082 | 6.804e-07 | 18 | 184765 | .000134 | 24.75338 |
| rs9435794 | C | .289 | .046 | .0094 | 9.198e-07 | 1 | 184765 | .0001296 | 23.94439 |
| rs7871607 | G | .0117 | .1982 | .0404 | 9.205e-07 | 9 | 149468 | .000161 | 24.06438 |
| rs205723 | A | .4141 | .041 | .0084 | 1.029e-06 | 7 | 184765 | .0001289 | 23.82063 |
| rs60369116 | G | .9754 | .1428 | .0295 | 1.241e-06 | 7 | 149468 | .0001567 | 23.42849 |
| rs11902472 | A | .6168 | .0405 | .0084 | 1.312e-06 | 2 | 184765 | .0001258 | 23.24325 |
| rs4377758 | G | .0561 | .0943 | .0195 | 1.339e-06 | 6 | 184765 | .0001266 | 23.38294 |
| rs79294243 | C | .0441 | .0989 | .0206 | 1.639e-06 | 2 | 184765 | .0001247 | 23.04644 |
| rs10849982 | G | .8283 | .052 | .0109 | 1.762e-06 | 12 | 184765 | .0001232 | 22.75623 |
| rs2059730 | G | .6686 | .0419 | .0088 | 1.854e-06 | 2 | 184765 | .0001227 | 22.6678 |
| rs11209802 | C | .6795 | .042 | .0088 | 1.957e-06 | 1 | 184765 | .0001233 | 22.77612 |
| rs146752096 | T | .0862 | .0691 | .0145 | 1.960e-06 | 2 | 184765 | .0001229 | 22.70736 |
| rs1012534 | A | .5662 | .0388 | .0082 | 2.251e-06 | 6 | 184765 | .0001212 | 22.38634 |
| rs1885331 | T | .7508 | .045 | .0095 | 2.333e-06 | 6 | 184765 | .0001214 | 22.43495 |
| rs2305758 | T | .2802 | .0425 | .009 | 2.699e-06 | 19 | 184765 | .0001207 | 22.29669 |
| rs78698099 | G | .9496 | .1008 | .0215 | 2.779e-06 | 16 | 149468 | .000147 | 21.9776 |
| rs13123620 | A | .5914 | .0387 | .0083 | 3.196e-06 | 4 | 184765 | .0001177 | 21.73775 |
| rs79777905 | G | .9824 | .1716 | .0369 | 3.315e-06 | 8 | 149468 | .0001447 | 21.62315 |
| rs9855698 | G | .1398 | .0564 | .0122 | 3.728e-06 | 3 | 184765 | .0001157 | 21.3692 |
| rs1587858 | C | .301 | .042 | .0091 | 3.786e-06 | 5 | 184765 | .0001153 | 21.29932 |
| rs1503510 | C | .6503 | .0397 | .0086 | 3.939e-06 | 5 | 184765 | .0001153 | 21.30758 |
| rs9655332 | T | .4229 | .0668 | .0145 | 3.953e-06 | 7 | 57980 | .0003659 | 21.21573 |
| rs701802 | G | .6396 | .0412 | .009 | 4.135e-06 | 10 | 184765 | .0001134 | 20.95367 |
| rs7513688 | G | .6418 | .0394 | .0086 | 4.480e-06 | 1 | 184765 | .0001136 | 20.9868 |
| rs2086512 | A | .1093 | .0596 | .013 | 4.552e-06 | 6 | 184765 | .0001137 | 21.01631 |
| rs73262787 | G | .9468 | .0861 | .0188 | 4.626e-06 | 12 | 184765 | .0001135 | 20.97207 |
| rs6948053 | G | .056 | .0856 | .0187 | 4.660e-06 | 7 | 184765 | .0001134 | 20.9515 |
| rs830147 | G | .9471 | .1703 | .0372 | 4.725e-06 | 19 | 57980 | .0003613 | 20.95012 |
| rs4837004 | C | .3402 | .0394 | .0086 | 4.787e-06 | 9 | 184765 | .0001136 | 20.9868 |
| rs4147187 | T | .021 | .1434 | .0314 | 4.952e-06 | 9 | 149468 | .0001395 | 20.85347 |
| rs149434117 | T | .9829 | .1642 | .036 | 4.962e-06 | 3 | 149468 | .0001392 | 20.80084 |
| rs62638743 | A | .0296 | .1157 | .0254 | 5.096e-06 | 19 | 184765 | .0001123 | 20.74677 |
| rs12949052 | A | .9258 | .073 | .016 | 5.103e-06 | 17 | 184765 | .0001127 | 20.81406 |
| rs6047198 | C | .7527 | .0433 | .0095 | 5.409e-06 | 20 | 184765 | .0001124 | 20.77207 |
| rs11724871 | C | .4671 | .0378 | .0083 | 5.766e-06 | 4 | 184765 | .0001122 | 20.73856 |
| rs7969834 | G | .273 | .0413 | .0092 | 6.578e-06 | 12 | 184765 | .0001091 | 20.15009 |
| rs10012797 | A | .9167 | .0684 | .0152 | 6.631e-06 | 4 | 184765 | .0001096 | 20.24778 |
| rs143529057 | C | .989 | .2036 | .0453 | 6.891e-06 | 5 | 149468 | .0001351 | 20.19763 |
| rs17294232 | A | .5541 | .0377 | .0084 | 7.105e-06 | 2 | 184765 | .000109 | 20.1408 |
| rs61997596 | A | .1862 | .0484 | .0108 | 7.111e-06 | 14 | 184765 | .0001087 | 20.08149 |
| rs79563551 | C | .9778 | .1427 | .0319 | 7.499e-06 | 4 | 149468 | .0001339 | 20.00822 |
| rs17481131 | T | .7925 | .0452 | .0101 | 8.062e-06 | 12 | 184765 | .0001084 | 20.02567 |
| rs139621111 | C | .1961 | .0877 | .0197 | 8.150e-06 | 7 | 57980 | .0003417 | 19.81154 |
| rs7670670 | T | .2236 | .0444 | .0099 | 8.177e-06 | 4 | 184765 | .0001089 | 20.11168 |
| rs80144387 | T | .0699 | .0857 | .0193 | 8.757e-06 | 2 | 162082 | .0001216 | 19.71488 |
| rs2049824 | C | .4674 | .0365 | .0082 | 9.076e-06 | 8 | 184765 | .0001072 | 19.81123 |
| rs10849767 | T | .3344 | .0397 | .009 | 9.384e-06 | 12 | 184765 | .0001053 | 19.45585 |
| rs72798040 | T | .9009 | .0624 | .0141 | 9.397e-06 | 16 | 184765 | .000106 | 19.58326 |
| rs4445597 | T | .1045 | .0603 | .0136 | 9.856e-06 | 11 | 184765 | .0001064 | 19.6567 |
| rs7758880 | C | .7075 | .036 | .0092 | .00008703 | 6 | 184765 | .0000829 | 15.31064 |

We computed for each SNP the variance (R^2^) and the F-statistic, as explained in Supplementary Methods.

None of the 65 SNPs were in linkage disequilibrium using LDpair tool (<https://ldlink.nih.gov/?tab=ldpair> [4]) when tested pairs by pairs among the same chromosome with a maximal R^2^ threshold < 0.2.

In red, SNPs excluded because of an insufficient significant p-value. In blue, SNPs not found in CAD-GWAS summary statistics

Chr, chromosome; EAF, effect allele frequency; SE, standard error. Beta coefficient corresponds to the log odds of ever use of cannabis. SNP, single-nucleotide polymorphism; EA, effect allele; EAF, effect allele frequency; Beta, the per-allele effect on cannabis use from the meta-analysis; Standard error of Beta; p-value is for the genetic association in the GWAS.; Sample size, depending on how many samples this SNP was present.

**Supplementary Table 3.** Summary of the observational studies included in the analysis of cannabis use and risk of ASCVD, presented by specific outcome and type of study design

| **Type of study (country, years of recruitment)** | **Years of follow-up** | **Cases*** | **Controls** | **Exposure (as reported in the text)** | **Outcome (source)** | **Adjustments** | **Reference (PMID)** | |
| --- | --- | --- | --- | --- | --- | --- | --- | --- |
| **Coronary artery disease including acute myocardial infarction** | | | | | | | |  |
| Cohort prospective (USA; 1985-1986) | 27 | 104 | 5009 | Cumulative marijuana use (never vs. min 0.5 marijuana-years) | CHD (telephone interviews, examinations, medical records) | Age, gender, race, education, family history of ASCVD, physical activity, BMI, HTA, DM, dyslipidemia, depression, smoking, cumulative alcohol use, cumulative use of other illicit drugs | Reis et al[5] 2017 (28207342) | |
| Cohort prospective (Swiss, 2003-2006) | 13 | 227 | 4112 | Cannabis use (never vs. abuse or dependence) | AMI and CHD (Electronic records with ICD codes) | Age, sex, smoking, BMI, hypertension, diabetes | Colaus/PsyCoLaus study (See Supplementary Methods) | |
| Cohort retrospective (USA, 2011-2016) | 3 | 60940 | 10'774’178 | History of cannabis abuse (ever v. never) | AMI (from multi-institutional database) | Age, sex, hypertension, coronary artery disease, diabetes, and other substance abuse | Chami et al[6], 2019 (31378243) | |
| A case -matched cohort study (USA, 2016-2018) | 2 | 84 | 465 | Cannabis use (never vs. use, abuse or dependence) | CHD (Electronic ICD-10^th^ codes) | BMI, smoking, households, other substances abuse, any chronic health condition, any acute health events | Philips et al[7], 2022 (35279458) | |
| Case-control study (USA, 2010-2014) | - | 1694 | 9'465’255 | Cannabis use (never vs. current cannabis use disorder) | AMI (Electronic records with ICD-codes) | Demographics, medical risk factors, and other substances abuse | Patel et al[8] 2020 (31611137**)** | |
| Case-control study (USA 2016-2017) | - | 201 | 1219 | Marijuana use (never vs. ever) | CHD according to CT angiogram | Age, DM, HTA | Burt et al[9] 2020 (31995626) | |
| **Ischemic stroke and transient ischemic attack** | | | | | | | |  |
| Cohort prospective (Sweden, 1949-1951) | 26 | 1037 | 44044 | Cannabis use (never vs. 1 to 10 times) | IS and TIA (Electronic records with ICD-8^th^ and -9^th^ codes) | Age, BMI, migraine, DM, family history of ASCVD, HTA, cardiorespiratory fitness, childhood socioeconomic position, short schooling, smoking, alcohol consumption | Falksted et al[10], 2017 (28028147) | |
| Cohort prospective (USA; 1985-1986) | 27 | 62 | 5051 | Cumulative marijuana use (never vs. min 0.5 marijuana-years) | IS and TIA (telephone interviews, examinations, medical records) | Age, gender, race, education, family history of ASCVD, physical activity, BMI, HTA, DM, dyslipidemia, depression, smoking, cumulative alcohol use, cumulative use of other illicit drugs | Reis et al[5] 2017 (2807342) | |
| Cohort prospective (Swiss, 2003-2006) | 13 | 79 | 4260 | Cannabis use (never vs. abuse or dependence) | IS and TIA (Electronic records with ICD codes) | Age, sex, smoking, BMI, hypertension, diabetes | CoLaus/PsyColaus study (See Supplementary Methods) | |
| Case-control study (USA 2004-2011) | - | 478’650 | 118’180’969 | Marijuana use (never vs. cannabis use disorders) | IS and vasospam (Electronic records with ICD-9^th^ codes) | Age, sex, race, payer status, Charlson’s comorbidity index, substances abuse | - Rumalla et al[11], 2016 (26874461) | |
| Cross-sectional study (USA, 2000-2003) | - | 998 | 811’249 | Cannabis use (ever vs. cannabis abuse or dependence) | IS (Electronic records with ICD-9^th^ codes) | Others addictive substances, cardiovascular comorbidities | - Westover et al[12], 2007 (17404126) | |
| Cross-sectional study (USA, 2007-2011) | - | 106957 | 3’949’000 | Cannabis use (ever vs. cannabis abuse or dependence) | IS (Electronic records with ICD-9^th^ codes) | Age, gender, race, residential income, insurance, residential region, pain, and number of comorbidities | - Vin Raviv - et al[13],2017 (27891823) | |
| **ASCVD mortality or all ASCVD** | | | | | | | |  |
| Cohort prospective (USA, 2005-2014) | 6 | 39 | 14818 | Cannabis use (never vs. ever) | ASCVD mortality^a^ including heart disease and cerebrovascular disease (Electronic records with ICD codes) | Age, sex, race/ethnicity, education, family income level, smoking, alcohol intake, physical activity, diet, BMI | Sun et al[14] 2020 (33220757) | |
| Cohort prospective  (USA; 1985-1986) | 27 | 215 | 4898 | Cumulative marijuana use (never vs. min 0.5 marijuana-years) | ASCVD mortality^b^ (telephone interviews, examinations, medical records) | Age, gender, race, education, family history of ASCVD, physical activity, BMI, HTA, DM, dyslipidemia, depression, smoking, cumulative alcohol use, cumulative use of other illicit drugs | Reis et al[5] 2017 (28207342) | |
| Cohort prospective (Canada, 1989) | 20 | 63923 | 1’247’035 | Cannabis use (never vs. current or past history of cannabis use disorders) | ASCVD hospitalizations^c^ (Electronic records with ICD codes) | Age, gravidity, mental illness, smoking, comorbidity, socioeconomic deprivation, place of residence, time-period | Auger et al[15] 2020 (33208143) | |
| Cohort prospective (Swiss, 2003-2006) | 12 | 299 | 4040 | Cannabis use (never vs. abuse or dependence) | ASCVD^d^ (Electronic records with ICD codes) | Age, sex, smoking, BMI, hypertension, diabetes | CoLaus/PsyCoLaus study (See Supplementary Methods) | |

AMI, acute myocardial infarction ; CHD, coronary heart disease; BMI, body mass index; HTA, hypertension arterial; IS, acute ischemic stroke; TIA, transient ischemic attack.

* cases involving participants with outcome event.

^a^Heart diseases included mainly diseases of the heart and blood vessels and hypertensive heart disease using NCHS codes I00–I09, I11, I13, I20–I51. Cerebrovascular diseases included mainly hemorrhagic and non-hemorrhagic stroke, as well as other cerebrovascular disease using NCHS codes I60–I69

^b^ASCVD definition was not provided in the original study, other than cardiovascular disease mortality.

^c^ASCVD hospitalization included heart disease, pulmonary vascular disease. cerebrovascular disease, hypertension, atherosclerosis, aortic aneurysm or dissection, aneurysm of other vessels, and arterial embolism using ICD-9/10 codes.

^d^ASCVD, comprised non-fatal acute myocardial infarction (AMI), symptomatic coronary artery disease with greater than 50% stenosis treated by percutaneous coronary intervention or coronary artery bypass graft (CHD), and fatal and non-fatal ischaemic stroke (including transient ischaemic attack).

**Supplementary Table 4.** Summary of the observational studies excluded in the analysis of cannabis use and risk of ASCVD, presented by specific outcome and type of study

| **Type of study (country, years of recruitment)** | **Reason of exclusion** | **Years of follow-up** | **Exposure** | **Outcome (source)** | **Adjustments (results: RR, 95% CI)** | **Reference (PMID)** | |
| --- | --- | --- | --- | --- | --- | --- | --- |
| **Coronary artery disease including acute myocardial infarction** | | | | | | |  |
| Cohort retrospective (USA,-) | Comparison group without never use | 7,5 | Cannabis use (non-frequent or never vs. frequent) | AMI (Electronic records with ICD-9^th^ and -10^th^ codes) | Other substance use disorders, time since the earliest observation of cannabis use, age, sex, race, ethnicity, median income, BMI **(OR, 1.25, 0.80 – 1.95)** | - Winhusen et al 2019 - (31743053) | |
| **Ischemic stroke and transient ischemic attack** | | | | | | |  |
| Cohort retrospective (USA,-) | Comparison group without never use | 7,5 | Cannabis use (non-frequent or never vs. frequent) | AMI (Electronic records with ICD-9^th^ and -10^th^ codes) | Other substance use disorders, time since the earliest observation of cannabis use, age, sex, race, ethnicity, median income, BMI **(OR, 1.62, 1.05–2.50)** | - Winhusen et al 2019 - (31743053) | |
| Cross-sectional study (Australia, 1999-2001) | Lifetime use of cannabis not available | - | Marijuana user (never vs. recent marijuana user) | Self-reported IS or TIA | Age, level of education, working status, smoking, HTA, DM, exercise frequency **(OR, 2.3, 1.1-4.5)** | Hemachandra et al 2016 (26558539) | |
| Cross-sectional study (USA, 2009-2010) | Imprecise outcome | - | Cannabis use (ever vss. Cannabis use disorders) | Cerebrovascular accident | Age, sex, HTA, DM, hyperlipidemia, CAD, smoking, and alcohol use  **(OR 1.24, 1.14-1.34)** | Kalla et al 2018 (29879084) | |
| Cross-sectional study (USA, 2016-2017) | Lifetime use of cannabis not available | - | Marijuana user (never vs. recent marijuana user) | Self-reported IS | Age, sex, race, education, marital status, BMI, physical activity, smoking, e-cigarette use, heavy drinking, DM  **(OR 1.82, 1.08–3.10)** | Parekh et al 2020 (31707926) | |
| **ASCVD mortality or all ASCVD** | | | | | | |  |
| Cohort prospective (USA, 1989-1994) | Lifetime use of cannabis not available | 4 | Marijuana use (never vs. recent use [1 year before inclusion]) | ASCVD mortality (death certificates from state offices of vital records) | Age and sex  **(HR 1.9, 0.6−6.3)** | - Mukamal et al, 2008 - ([18294478](https://pubmed.ncbi.nlm.nih.gov/18294478)) | |
| Cohort prospective (USA, 2000-2016) | Lifetime use of cannabis not available | 11 | Marijuana use (never vs. recent use [one week before inclusion]) | ASVCD mortality (Electronic records with ICD codes) | Age, sex, DM, HTA, peripheral vascular disease, smoking, HDL-C, triglycerides, revascularization, creatinine, medications at discharge, length of stay  **(HR 2.09, 1.25- 3.5)** | DeFilipis et al, 2018 (29535062) | |
| Cohort prospective (USA, 1990-2010) | Comparison group without never use | 6 | Heavy cannabis users (occasional use or nonuse vs. daily or weekly use | ASCVD (Electronic record with ICD-9^th^ codes) | Age, smoking, viral load, traditional cardiovascular risk factor  **(HR 2.16 1.04-4.51)** | [Lorenz](#_ENREF_6) et al 2017 (28449059) | |

AMI, acute myocardial infarction ; CHD, coronary heart disease; BMI, body mass index; HTA, hypertension arterial; IS, acute ischemic stroke; TIA, transient ischemic attack.

**Supplementary Table 5**. Power (two-sided α=0.05) for conventional Mendelian randomization analysis

|  | **Exposure** | **Actual N in outcome-GWAS** | **Proportion of cases in outcome-GWAS** | **Observational OR** | **R2 of instrument** | **N required for 80% power** | **Power at actual N** |
| --- | --- | --- | --- | --- | --- | --- | --- |
| **For CAD** | **Cannabis use** | 184’305 | 0.330 | 1.23* | 0.01 | 76895 | 0.99 |
| **For IS** | **Cannabis use** | 440328 | 0.084 | 1.22* | 0.01 | 217837 | 0.98 |

Power calculation was based on the method developed by Brion et al.[^13^](#_ENREF_13) <https://shiny.cnsgenomics.com/mRnd/> * from observational meta-analysis for CAD and IS respectively (Figure 1).

**Supplementary Table 6.** Comparison of conventional MR, MR-Egger, MR-Egger adjusted for SIMEX and weighted median MR causal effect estimates of cannabis use on risk of coronary artery disease

| **Analysis** | **Causal effect estimate** | **95% CI** |
| --- | --- | --- |
| **Conventional MR** | -0.03 | -0.09-0.03 |
| **MR-Egger** (*I^2^*=0.18) | -0.01 | -0.13; 0.11 |
| **MR-Egger+SIMEX** | -0.03 | -0.13- 0.08 |
| **Weighted median MR** | -0.05 | -0.13; 0.03 |

Estimates are expressed as Log OR per-1-log unit increase in ever use of cannabis. Conventional MR was pooled effect across SNPs using fixed-effect with inverse variance weighted meta-analysis. As previously described by Bowden et al [16], it is noteworthy to mention that power to detect a causal effect using MR-Egger analysis is largely underpowered (as shown by the corresponding large confidence intervals) with the use of 63 SNPs. The adjusted MR-Egger regression estimate (derived by simulation extrapolation [SIMEX] to account for a potential violation of the NOME assumption) is the result of 100,000 simulations. *I^2^* quantifies weak instrument bias in the context of MR-Egger. *for significant p-value (p-value<0.05)

**Supplementary Table 7.** Comparison of conventional MR, MR-Egger, MR-Egger adjusted for SIMEX and weighted median MR causal effect estimates of cannabis use on risk of acute ischemic stroke

| **Analysis** | **Causal effect estimate** | **95% CI** |
| --- | --- | --- |
| **Conventional MR** | 0.08 | -0.05-0.10 |
| **MR-Egger** (*I^2^*=0.39) | 0.04 | -0.11-0.18 |
| **MR-Egger+SIMEX** | -0.01 | -0.15-0.12 |
| **Weighted median MR** | 0.02 | -0.07-0.11 |

Estimates are expressed as Log OR per-1-log unit increase in ever use of cannabis. Conventional MR was pooled effect across SNPs using fixed-effect with inverse variance weighted meta-analysis. As previously described by Bowden et al, it is noteworthy to mention that power to detect a causal effect using MR-Egger analysis is largely underpowered (as shown by the corresponding large confidence intervals) with the use of 64 SNPs. The adjusted MR-Egger regression estimate (derived by simulation extrapolation [SIMEX] to account for a potential violation of the NOME assumption) is the result of 100,000 simulations. *I^2^* quantifies weak instrument bias in the context of MR-Egger. *for significant p-value (p-value<0.05)

**Supplementary Table 8.** Comparison of conventional MR, MR-Egger and weighted median MR causal effect estimates of cannabis use on ASVCD restricted to 5 SNPs with p-value<5x10^-8^

|  | **Coronary artery disease** | | **Acute ischemic stroke** | |
| --- | --- | --- | --- | --- |
| **Analysis** | **Causal effect estimate** | **95% CI** | **Causal effect estimate** | **95% CI** |
| **Conventional MR** | -0.13 | -0.26-0.01 | 0.03 | -0.12-0.17 |
| **MR-Egger** | -0.07 | -0.41-0.27 | -0.12 | -0.47-0.22 |
| **Weighted median MR** | -0.12 | -0.28-0.05 | -0.01 | -0.19-0.16 |

Estimates are expressed as Log OR per-1-log unit increase in ever use of cannabis. Conventional MR was pooled effect across SNPs using fixed-effect with inverse variance weighted meta-analysis. No evidence against the null hypothesis of no directional pleiotropy of the genetic markers using MR-Egger was found (P-value for pleiotropy=0.730 for CAD and = 0.346 for IS). *for significant p-value (p-value<0.05)

**Supplementary Table 9.** Comparison of conventional MR, MR-Egger and weighted median MR causal effect estimates of cannabis use on ASVCD with removing SNPs being palindromic with intermediate allele frequencies.

|  | **Coronary artery disease** | | | | **Acute ischemic stroke** | | | |
| --- | --- | --- | --- | --- | --- | --- | --- | --- |
|  | **64 SNPs (p-value<5x10^-5^)** | | **4 SNPs (p-value<5x10^-8^)** | | **65 SNPs (p-value<5x10^-5^)** | | **4 SNPs (p-value<5x10^-8^)** | |
| **Analysis** | **Causal effect estimate** | **95% CI** | **Causal effect estimate** | **95% CI** | **Causal effect estimate** | **95% CI** | **Causal effect estimate** | **95% CI** |
| **Conventional MR** | -0.03 | -0.08-0.02 | -0.13 | -0.31-0.05 | 0.03 | -0.04-0.10 | 0.10 | -0.08-0.28 |
| **MR-Egger** | -0.02 | -0.14-0.12 | 0.10 | -0.87-1.06 | 0.04 | -0.13-0.22 | 0.17 | -1.06-1.40 |

We cannot verify that alleles reported by CardioGRAMplusC4D or MEGASTROKE have been correctly orientated, therefore we removed palindromic SNPs if the allele frequency was close to 50%. Estimates are expressed as Log OR per-1-log unit increase in ever use of cannabis. Conventional MR was pooled effect across SNPs using fixed-effect with inverse variance weighted meta-analysis. No evidence against the null hypothesis of no directional pleiotropy of the genetic markers using MR-Egger intercept was found for overall and restricted SNPs (P-value for pleiotropy=0.753 and =0.684 for CAD; p-value=0.855 and =0.921 for IS, respectively). *for significant p-value (p-value<0.05)

**Supplementary Table 10.** Comparison of conventional MR, MR-Egger and weighted median MR causal effect estimates of cannabis use on ASCVD using cannabis use disorder as modifiable exposure

|  | **Coronary artery disease** | | **Acute ischemic stroke** | |
| --- | --- | --- | --- | --- |
|  | **103 SNPs (p-value<5x10^-5^)** | | **106 SNPs (p-value<5x10^-5^)** | |
| **Analysis** | **Causal effect estimate** | **95% CI** | **Causal effect estimate** | **95% CI** |
| **Conventional MR** | 0.02 | -0.002-0.04 | 0.03 | -0.0004-0.06 |
| **MR-Egger** | 0.03 | -0.05-0.08 | 0.06 | -0.0006-0.12* |
| **Weighted median MR** | 0.007 | -0.03-0.04 | 0.02 | -0.01-0.06 |

We selected SNPs with p-value that were associated with cannabis use disorder (p-value<5x10^-5^) and in low linkage-disequilibrium with other SNPs (R^2^< 0.001) within a clumping distance of 10,000 kb. Palindromic SNPs with intermediate minor allele frequency were removed because we cannot assumed that all alleles were correctly reported in the positive strand in cannabis use disorder GWAS. A more restricted threshold with p-value <5x10^-8^ was initially computed but only 2 SNPs remained, which was not sufficient for two-sample mendelian randomization analysis. Estimates are expressed as Log OR per-1-log unit increase in ever use of cannabis. Conventional MR was pooled effect across SNPs using fixed-effect with inverse variance weighted meta-analysis. *for significant p-value (p-value<0.05). There was evidence of directional pleiotropy for IS with a p-value=0.048 using MR-Egger intercept (p-value=0.116 for CAD).

**Supplementary Table 11.** Excluded studies from systematic reviews literature research

| **Systematic reviews identified by the literature research** | **Reasons for exclusion** |
| --- | --- |
| PMID 28432636 | 81 case reports study  12 case series study  2 experimental studies  3 clinical trial without outcome of interest  1 forum discussion  4 descriptive analyses of exposure population only  8 with inappropriate outcome  2 with inappropriate exposure (trigger instead of lifetime use or other cannabis as co-variable) |
| PMID 29357394 | 1 study was retracted  2 with inappropriate exposure (trigger instead of lisetime use)  5 with inappropriate outcome |
| PMID 34001774 | 2 case series study  4 lack of association analysis or descriptive analysis without controls  4 with inappropriate outcome  1 with inappropriate exposure (trigger instead of lifetime) |
| PMID 33636088 | 4 descriptive study  2 with inappropriate exposure (trigger instead of lifetime use  3 with inappropriate outcome (Moon cardiovascular event leading surgical procedure) |

**Supplementary Table 12**. Literature research’ strategies

| **Steps of literature researchs** | **Exposure terms** | **Outcome terms** | **Others terms** |
| --- | --- | --- | --- |
| Research of meta-analysis | ("cannabis*" [MeSH] OR "marijuana*"[MeSH] OR "cannabinoids" OR "delta-9-tetrahydrocannabinol" OR “cannabidiol” OR “cannabinol” OR “sativex” OR “hash” OR “ganka” OR “weed” OR “hemp” OR “THC” OR “CBD”) | ("Cardiovascular Diseases" [MeSH] OR “Myocardial Infarction”[Mesh] or “Stroke” [Mesh] OR “Cardiac Disease” OR “Heart Disease” OR “Vascular Disease” OR “Acute myocardial ischemia” OR “Myocardial*” OR “Acute Coronary Syndrome” OR “Angor” OR “Coronary heart disease” OR “Cardiac Ischemia” OR “Atherosclerosis” OR “Cerebrovascular disease” OR “Ischemic transient attack” OR “Cardiac Arrest” OR “Heart Arrest” OR “Cardiovascular death”) | "humans"[MeSH]  AND  "Meta-Analysis" [Publication Type] |
| Reasearch of systematic reviews | Similar | Similar | "humans"[MeSH]  AND  "Systematic Review" [Publication Type] |
| Research for pospective or retrospectives observational studies* | Similar | Similar | "humans"[MeSH]  AND  ("Cohort Studies"[Mesh] OR "Proportional Hazards Models"[Mesh] OR “prospective” OR “retrospective”) |

*We used a Year timeline for this research from 2016 to august 2022, because the older systematic review included in our comprehensive literature analysis was in 2016.

**References**

1. Pasman JA, Verweij KJH, Gerring Z, Stringer S, Sanchez-Roige S, Treur JL, Abdellaoui A, Nivard MG, Baselmans BML, Ong JS, Ip HF, van der Zee MD, Bartels M, Day FR, Fontanillas P, Elson SL, 23andMe Research Team, de Wit H, Davis LK, MacKillop J, Substance Use Disorders Working Group of the Psychiatric Genomics Consortium, International Cannabis Consortium, Derringer JL, Branje SJT, Hartman CA, Heath AC, van Lier PAC, Madden PAF, Mägi R, Meeus W, Montgomery GW, Oldehinkel AJ, Pausova Z, Ramos-Quiroga JA, Paus T, Ribases M, Kaprio J, Boks MPM, Bell JT, Spector TD, Gelernter J, Boomsma DI, Martin NG, MacGregor S, Perry JRB, Palmer AA, Posthuma D, Munafò MR, Gillespie NA, Derks EM, Vink JM. GWAS of lifetime cannabis use reveals new risk loci, genetic overlap with psychiatric traits, and a causal influence of schizophrenia. Nat Neurosci. 2018 Sep;21(9):1161–70.

2. Nikpay M, Goel A, Won HH, Hall LM, Willenborg C, Kanoni S, Saleheen D, Kyriakou T, Nelson CP, Hopewell JC, Webb TR, Zeng L, Dehghan A, Alver M, Armasu SM, Auro K, Bjonnes A, Chasman DI, Chen S, Ford I, Franceschini N, Gieger C, Grace C, Gustafsson S, Huang J, Hwang SJ, Kim YK, Kleber ME, Lau KW, Lu X, Lu Y, Lyytikäinen LP, Mihailov E, Morrison AC, Pervjakova N, Qu L, Rose LM, Salfati E, Saxena R, Scholz M, Smith AV, Tikkanen E, Uitterlinden A, Yang X, Zhang W, Zhao W, de Andrade M, de Vries PS, van Zuydam NR, Anand SS, Bertram L, Beutner F, Dedoussis G, Frossard P, Gauguier D, Goodall AH, Gottesman O, Haber M, Han BG, Huang J, Jalilzadeh S, Kessler T, König IR, Lannfelt L, Lieb W, Lind L, Lindgren CM, Lokki ML, Magnusson PK, Mallick NH, Mehra N, Meitinger T, Memon F ur R, Morris AP, Nieminen MS, Pedersen NL, Peters A, Rallidis LS, Rasheed A, Samuel M, Shah SH, Sinisalo J, Stirrups KE, Trompet S, Wang L, Zaman KS, Ardissino D, Boerwinkle E, Borecki IB, Bottinger EP, Buring JE, Chambers JC, Collins R, Cupples LA, Danesh J, Demuth I, Elosua R, Epstein SE, Esko T, Feitosa MF, Franco OH, Franzosi MG, Granger CB, Gu D, Gudnason V, Hall AS, Hamsten A, Harris TB, Hazen SL, Hengstenberg C, Hofman A, Ingelsson E, Iribarren C, Jukema JW, Karhunen PJ, Kim BJ, Kooner JS, Kullo IJ, Lehtimäki T, Loos RJF, Melander O, Metspalu A, März W, Palmer CN, Perola M, Quertermous T, Rader DJ, Ridker PM, Ripatti S, Roberts R, Salomaa V, Sanghera DK, Schwartz SM, Seedorf U, Stewart AF, Stott DJ, Thiery J, Zalloua PA, O’Donnell CJ, Reilly MP, Assimes TL, Thompson JR, Erdmann J, Clarke R, Watkins H, Kathiresan S, McPherson R, Deloukas P, Schunkert H, Samani NJ, Farrall M, the CARDIoGRAMplusC4D Consortium. A comprehensive 1000 Genomes–based genome-wide association meta-analysis of coronary artery disease. Nat Genet. 2015 Oct;47(10):1121–30.

3. Malik R, Chauhan G, Traylor M, Sargurupremraj M, Okada Y, Mishra A, Rutten-Jacobs L, Giese AK, van der Laan SW, Gretarsdottir S, Anderson CD, Chong M, Adams HHH, Ago T, Almgren P, Amouyel P, Ay H, Bartz TM, Benavente OR, Bevan S, Boncoraglio GB, Brown RD, Butterworth AS, Carrera C, Carty CL, Chasman DI, Chen WM, Cole JW, Correa A, Cotlarciuc I, Cruchaga C, Danesh J, de Bakker PIW, DeStefano AL, den Hoed M, Duan Q, Engelter ST, Falcone GJ, Gottesman RF, Grewal RP, Gudnason V, Gustafsson S, Haessler J, Harris TB, Hassan A, Havulinna AS, Heckbert SR, Holliday EG, Howard G, Hsu FC, Hyacinth HI, Ikram MA, Ingelsson E, Irvin MR, Jian X, Jiménez-Conde J, Johnson JA, Jukema JW, Kanai M, Keene KL, Kissela BM, Kleindorfer DO, Kooperberg C, Kubo M, Lange LA, Langefeld CD, Langenberg C, Launer LJ, Lee JM, Lemmens R, Leys D, Lewis CM, Lin WY, Lindgren AG, Lorentzen E, Magnusson PK, Maguire J, Manichaikul A, McArdle PF, Meschia JF, Mitchell BD, Mosley TH, Nalls MA, Ninomiya T, O’Donnell MJ, Psaty BM, Pulit SL, Rannikmäe K, Reiner AP, Rexrode KM, Rice K, Rich SS, Ridker PM, Rost NS, Rothwell PM, Rotter JI, Rundek T, Sacco RL, Sakaue S, Sale MM, Salomaa V, Sapkota BR, Schmidt R, Schmidt CO, Schminke U, Sharma P, Slowik A, Sudlow CLM, Tanislav C, Tatlisumak T, Taylor KD, Thijs VNS, Thorleifsson G, Thorsteinsdottir U, Tiedt S, Trompet S, Tzourio C, van Duijn CM, Walters M, Wareham NJ, Wassertheil-Smoller S, Wilson JG, Wiggins KL, Yang Q, Yusuf S, AFGen Consortium, Cohorts for Heart and Aging Research in Genomic Epidemiology (CHARGE) Consortium, International Genomics of Blood Pressure (iGEN-BP) Consortium, INVENT Consortium, STARNET, Bis JC, Pastinen T, Ruusalepp A, Schadt EE, Koplev S, Björkegren JLM, Codoni V, Civelek M, Smith NL, Trégouët DA, Christophersen IE, Roselli C, Lubitz SA, Ellinor PT, Tai ES, Kooner JS, Kato N, He J, van der Harst P, Elliott P, Chambers JC, Takeuchi F, Johnson AD, BioBank Japan Cooperative Hospital Group, COMPASS Consortium, EPIC-CVD Consortium, EPIC-InterAct Consortium, International Stroke Genetics Consortium (ISGC), METASTROKE Consortium, Neurology Working Group of the CHARGE Consortium, NINDS Stroke Genetics Network (SiGN), UK Young Lacunar DNA Study, MEGASTROKE Consortium, Sanghera DK, Melander O, Jern C, Strbian D, Fernandez-Cadenas I, Longstreth WT, Rolfs A, Hata J, Woo D, Rosand J, Pare G, Hopewell JC, Saleheen D, Stefansson K, Worrall BB, Kittner SJ, Seshadri S, Fornage M, Markus HS, Howson JMM, Kamatani Y, Debette S, Dichgans M. Multiancestry genome-wide association study of 520,000 subjects identifies 32 loci associated with stroke and stroke subtypes. Nat Genet. 2018 Apr;50(4):524–37.

4. Machiela MJ, Chanock SJ. LDlink: a web-based application for exploring population-specific haplotype structure and linking correlated alleles of possible functional variants. Bioinformatics. 2015 Nov 1;31(21):3555–7.

5. Reis JP, Auer R, Bancks MP, Goff DC, Lewis CE, Pletcher MJ, Rana JS, Shikany JM, Sidney S. Cumulative Lifetime Marijuana Use and Incident Cardiovascular Disease in Middle Age: The Coronary Artery Risk Development in Young Adults (CARDIA) Study. Am J Public Health. 2017 Apr;107(4):601–6.

6. Chami T, Kim CH. Cannabis Abuse and Elevated Risk of Myocardial Infarction in the Young: A Population-Based Study. Mayo Clin Proc. 2019 Aug;94(8):1647–9.

7. Phillips KT, Pedula KL, Choi NG, Tawara KAK, Simiola V, Satre DD, Owen-Smith A, Lynch FF, Dickerson J. Chronic health conditions, acute health events, and healthcare utilization among adults over age 50 in Hawai’i who use cannabis: A matched cohort study. Drug Alcohol Depend. 2022 May 1;234:109387.

8. Patel RS, Manocha P, Patel J, Patel R, Tankersley WE. Cannabis Use Is an Independent Predictor for Acute Myocardial Infarction Related Hospitalization in Younger Population. J Adolesc Health. 2020 Jan;66(1):79–85.

9. Burt JR, Agha AM, Yacoub B, Zahergivar A, Pepe J. Marijuana use and coronary artery disease in young adults. PLoS One. 2020;15(1):e0228326.

10. Falkstedt D, Wolff V, Allebeck P, Hemmingsson T, Danielsson AK. Cannabis, Tobacco, Alcohol Use, and the Risk of Early Stroke: A Population-Based Cohort Study of 45 000 Swedish Men. Stroke. 2017 Feb;48(2):265–70.

11. Rumalla K, Reddy A, Mittal M. Recreational marijuana Use and acute ischemic stroke: A population-based analysis of hospitalized patients in the United States. Journal of the Neurological Sciences. 2016 Feb 1;364.

12. Westover AN, McBride S, Haley RW. Stroke in young adults who abuse amphetamines or cocaine: a population-based study of hospitalized patients. Arch Gen Psychiatry. 2007 Apr;64(4):495–502.

13. Vin-Raviv N, Akinyemiju T, Meng Q, Sakhuja S, Hayward R. Marijuana use and inpatient outcomes among hospitalized patients: analysis of the nationwide inpatient sample database. Cancer Med. 2017 Jan;6(1):320–9.

14. Sun Y, Liu B, Wallace RB, Bao W. Association of Cannabis Use With All-Cause and Cause-Specific Mortality Among Younger- and Middle-Aged U.S. Adults. Am J Prev Med. 2020 Dec;59(6):873–9.

15. Auger N, Paradis G, Low N, Ayoub A, He S, Potter BJ. Cannabis use disorder and the future risk of cardiovascular disease in parous women: a longitudinal cohort study. BMC Med. 2020 Nov 19;18(1):328.

16. Bowden J, Davey Smith G, Burgess S. Mendelian randomization with invalid instruments: effect estimation and bias detection through Egger regression. Int J Epidemiol. 2015 Apr;44(2):512–25.
