# Supplementary material for: Cannabis use and atherosclerotic cardiovascular disease: a Mendelian randomization study
Source: BMC Cardiovasc Disord. 2023 Dec 13;23:611. doi: 10.1186/s12872-023-03641-w (PMC10717446; doi:10.1186/s12872-023-03641-w)
Supplement: Supplementary file 3 — Additional file 3. Supplementary Figures. [file 12872_2023_3641_MOESM3_ESM.docx]

**Additional file 3 - Supplementary Figures**

**Supplementary Figure 1.** Venn diagram showing the number of individuals and the overlap between the cannabis-GWAS and the CAD-GWAS


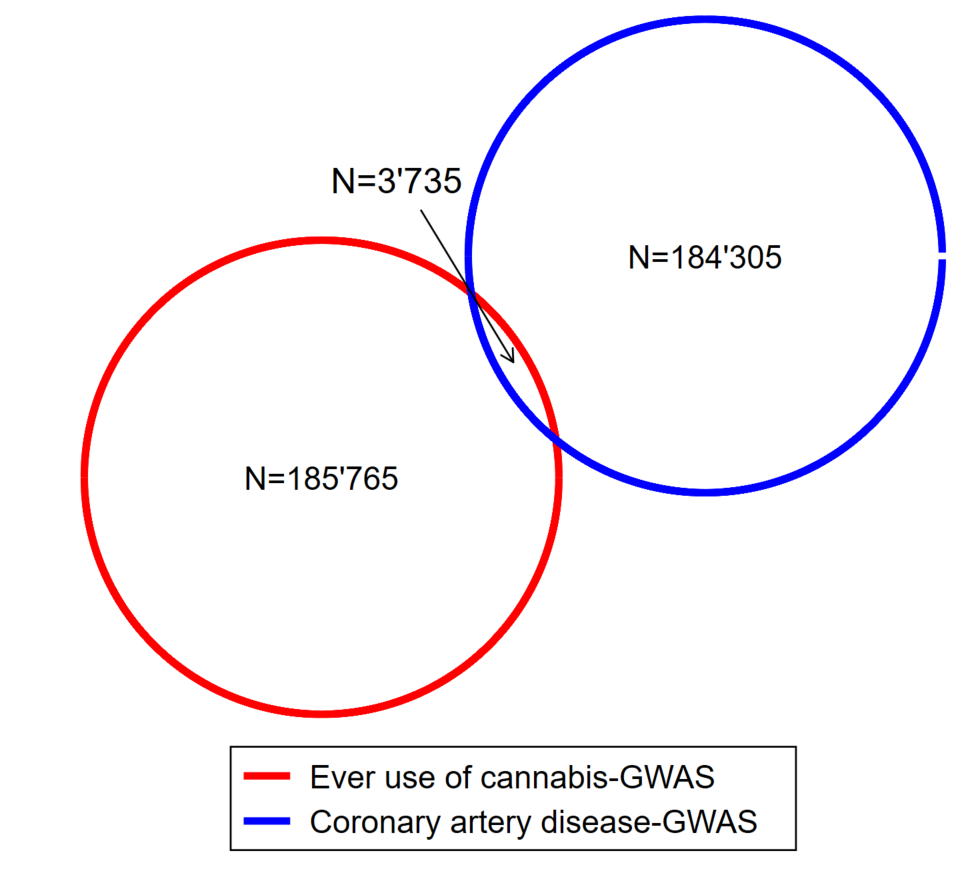


Based on the data provided in Pasman[1] et al and Nikpay et al[2], only the ECGUT study (Estonian Genome Center University of Tartu (N=3,735)) contributed to both GWAS.

**Supplementary Figure 2**. Flow chart of selection of studies included in our meta-analysis


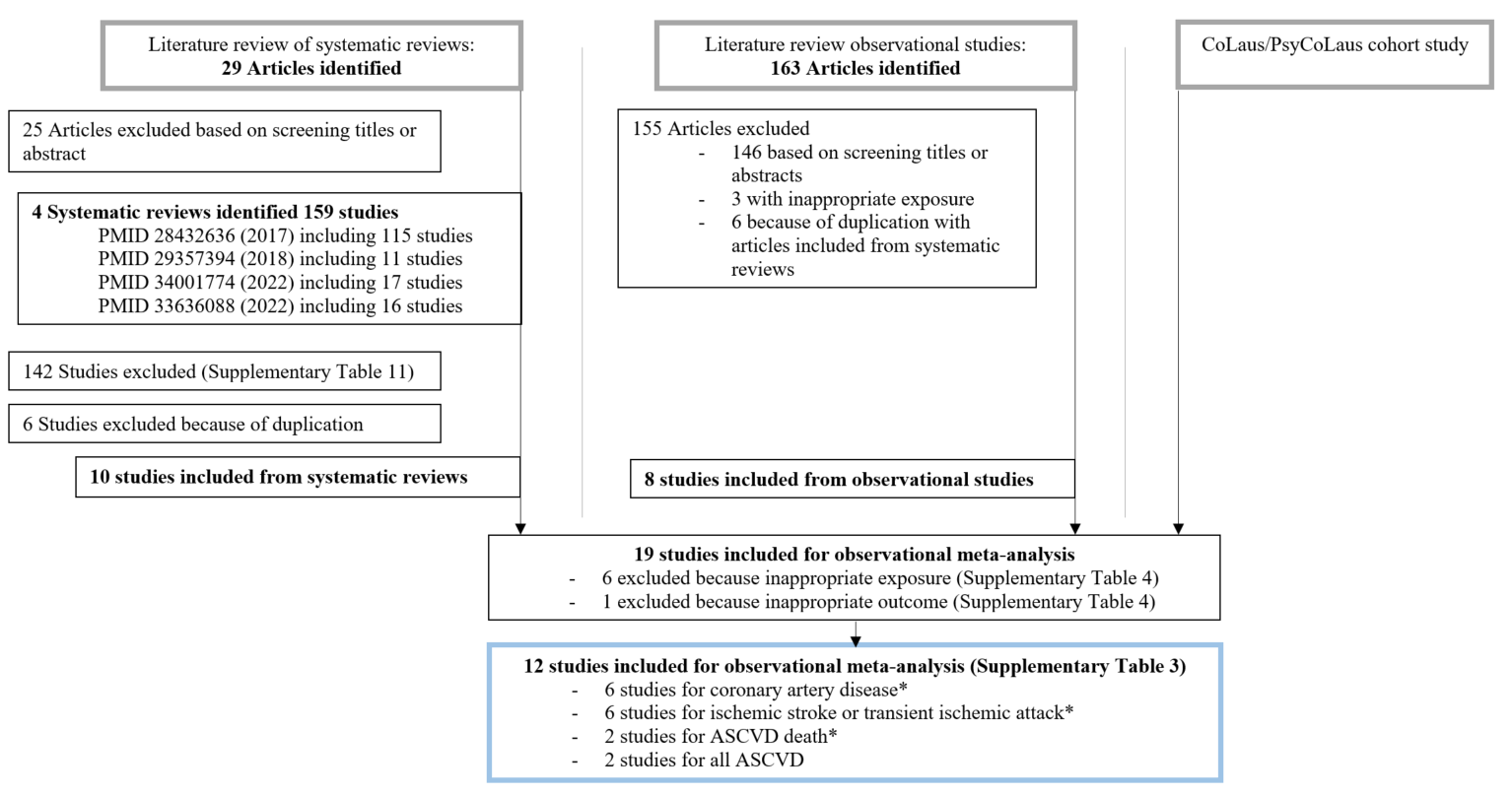


Details for research strategy can be found in Supplementary Table 12. *sum of subtotal studies are not equal to total studies because some studies accounts for different outcomes.

**Supplementary Figure 3**. Meta-analysis and forest plot of observational studies reporting an association between lifetime cannabis use and risk of coronary artery disease

Random-effect meta-analysis was calculated using DerSimonian and Laird methods (DL). Boxes are scaled according to the weight of the study in the random-effect model.

**Supplementary Figure 4**. Meta-analysis and forest plot of observational studies reporting an association between lifetime cannabis use and risk of ischemic stroke

Random-effect meta-analysis was calculated using DerSimonian and Laird methods (DL). Boxes are scaled according to the weight of the study in the random-effect model.

**Supplementary Figure 5**. Meta-analysis and forest plot of prospective observational studies reporting an association between lifetime cannabis use and risk of arteriosclerotic cardiovascular disease

Random-effect meta-analysis was calculated using DerSimonian and Laird methods (DL). Boxes are scaled according to the weight of the study in the random-effect model.

**Supplementary Figure 6**. Pair-wise association plot of the SNPs associated with cannabis use and risk of CAD


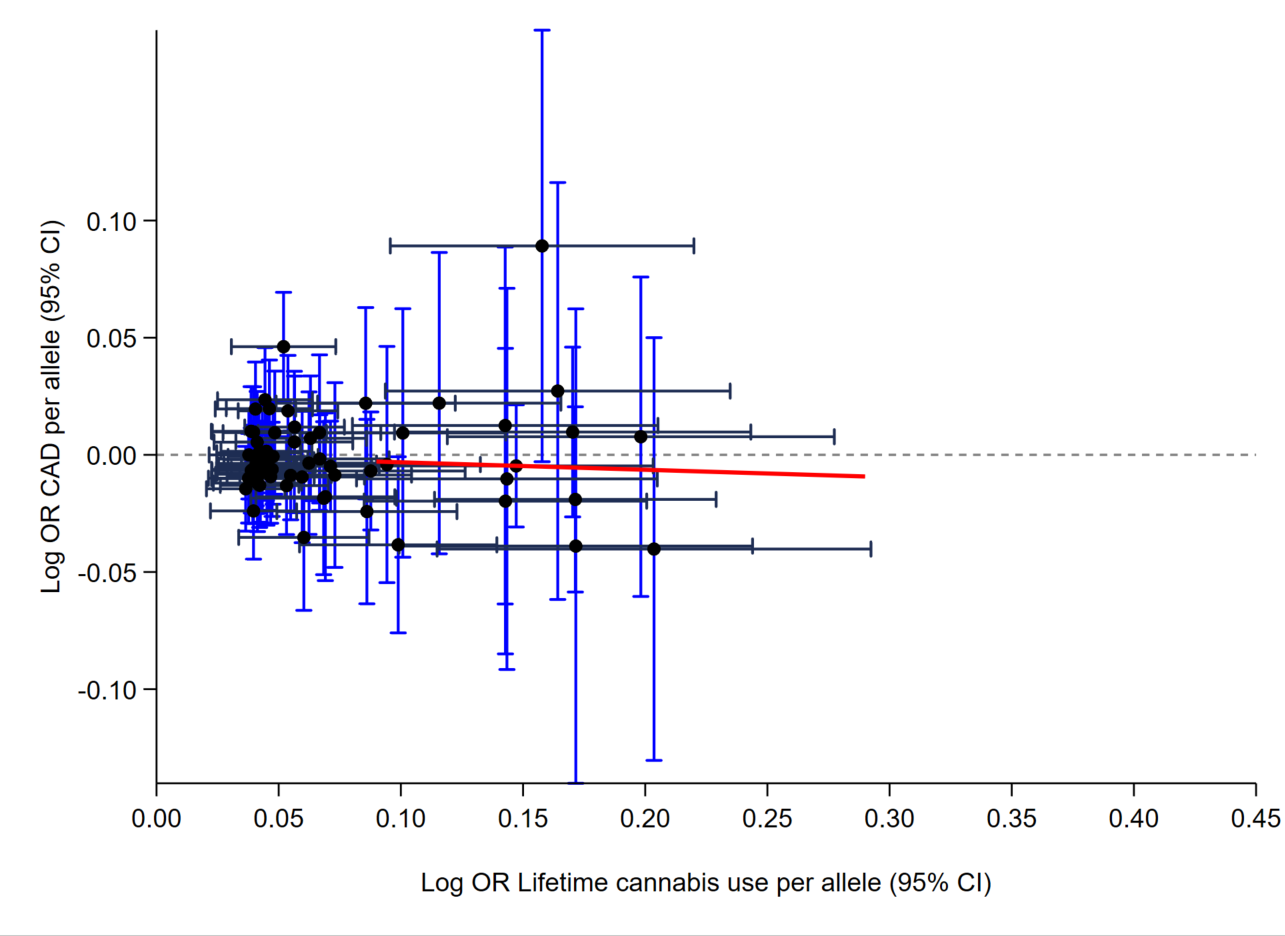


The red line represents the regression slope of the causal effects estimates (derived by the inverse-variance weighted approach as proposed by Bowden et al.).14

**Supplementary Figure 7**. Pair-wise association plot of the 10 SNPs associated with cannabis use and risk of IS


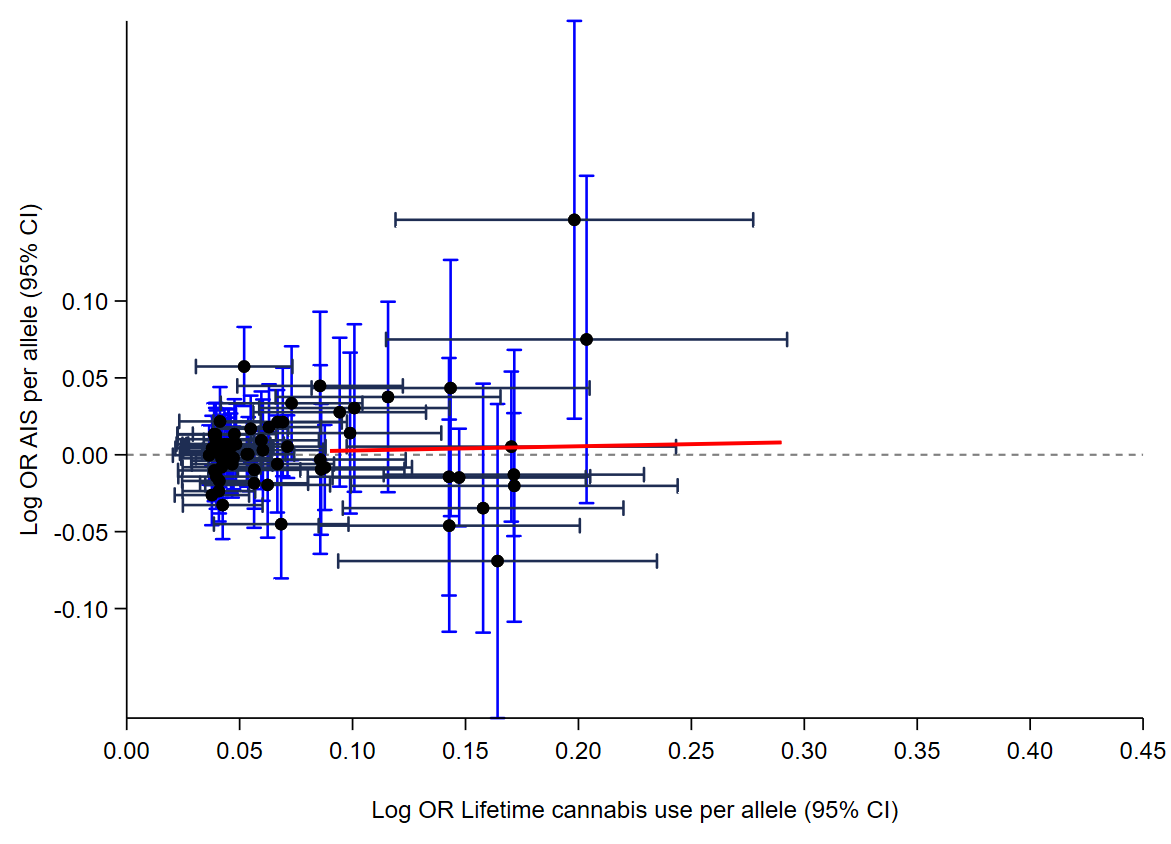


The red line represents the regression slope of the causal effects estimates (derived by the inverse-variance weighted approach as proposed by Bowden et al.).14

**Supplementary Figure 8.** Scatter plot of the genetic association with cannabis use against genetic association with coronary artery disease


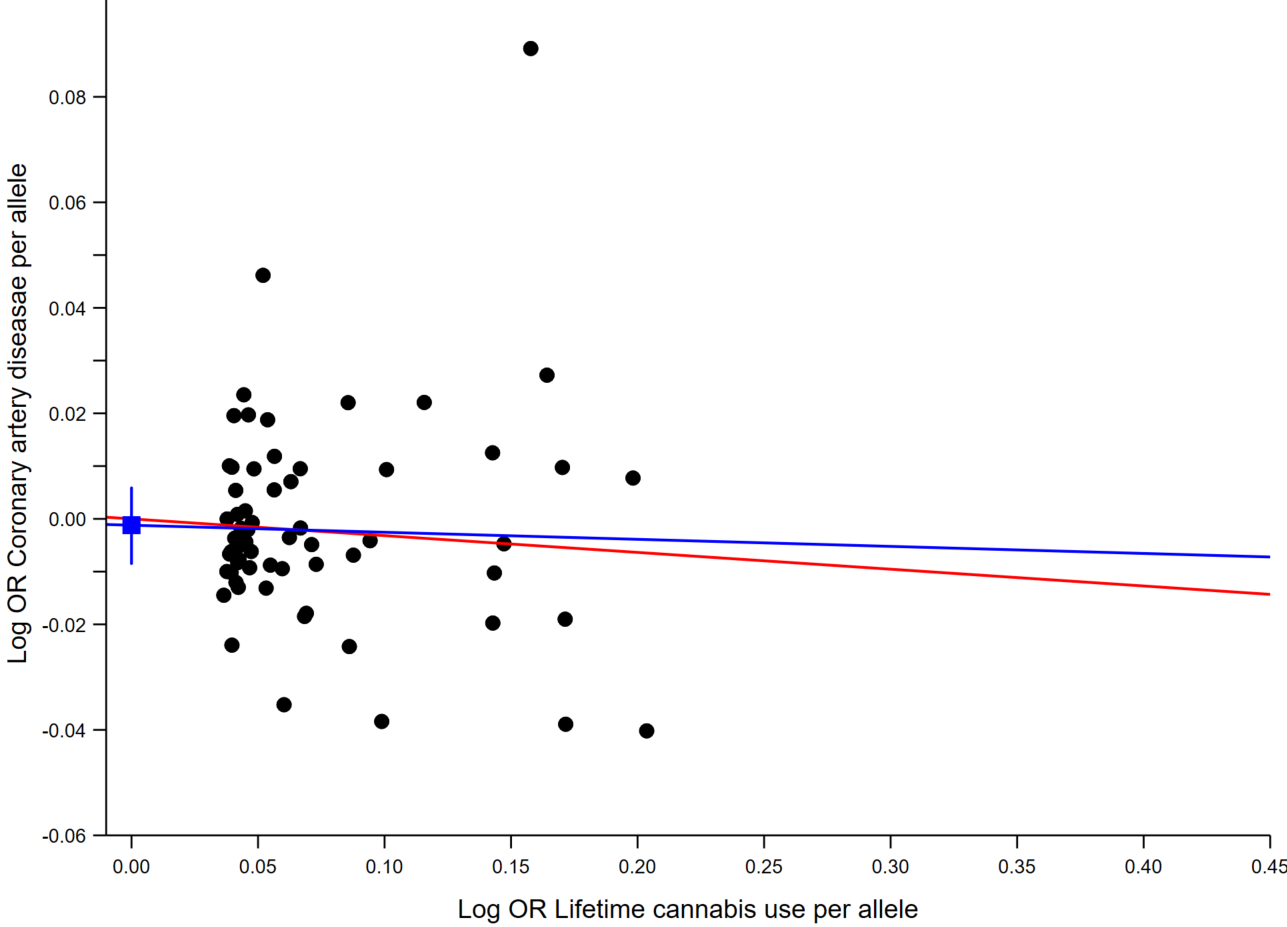


The conventional Mendelian randomization (Conventional MR in red), Egger Mendelian randomization (MR-Egger in blue) causal effects estimates are presented as regression slopes. The constant and its 95% CI (obtained by bootstrap resampling 10,000 times) derived from Egger regression are shown as the blue square and vertical bar, respectively.

**Supplementary Figure 9.** Scatter plot of the genetic association with cannabis use against genetic association with acute ischemic stroke


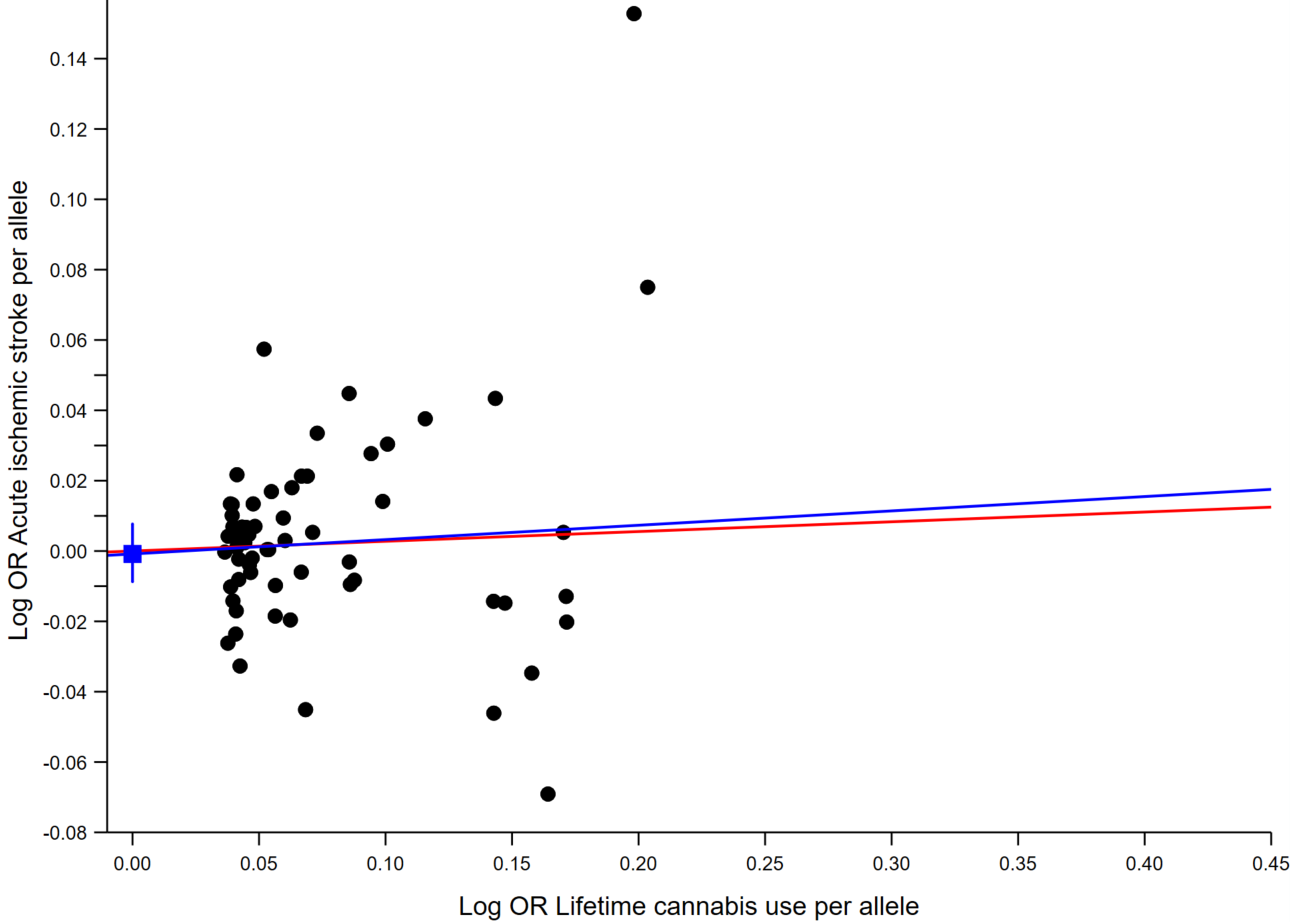


The conventional Mendelian randomization (Conventional MR in red), Egger Mendelian randomization (MR-Egger in blue) causal effects estimates are presented as regression slopes. The constant and its 95% CI (obtained by bootstrap resampling 10,000 times) derived from Egger regression are shown as the blue square and vertical bar, respectively.

Supplementary Figure 10. Meta-analysis of observational studies reporting an association between use of cannabis and risk of atherosclerotic cardiovascular disease without Sun et al study.

Meta-analysis without Sun et al study as part of the ASCVD sub-group. Studies are sorted by type of outcome (coronary artery disease, ischemic stroke or global ASCVD analysis). Relative risks (RR) and 95% confidence intervals (CI) express the risk of ASCVD for “ever use of cannabis” (compared with never use). For additional information on each study, see Supplementary Table 1. Supplementary Figure 4 and 5 provide meta-analysis stratified by outcome and type of risk ratio.

**Reference**

1. Pasman JA, Verweij KJH, Gerring Z, Stringer S, Sanchez-Roige S, Treur JL, Abdellaoui A, Nivard MG, Baselmans BML, Ong JS, Ip HF, van der Zee MD, Bartels M, Day FR, Fontanillas P, Elson SL, 23andMe Research Team, de Wit H, Davis LK, MacKillop J, Substance Use Disorders Working Group of the Psychiatric Genomics Consortium, International Cannabis Consortium, Derringer JL, Branje SJT, Hartman CA, Heath AC, van Lier PAC, Madden PAF, Mägi R, Meeus W, Montgomery GW, Oldehinkel AJ, Pausova Z, Ramos-Quiroga JA, Paus T, Ribases M, Kaprio J, Boks MPM, Bell JT, Spector TD, Gelernter J, Boomsma DI, Martin NG, MacGregor S, Perry JRB, Palmer AA, Posthuma D, Munafò MR, Gillespie NA, Derks EM, Vink JM. GWAS of lifetime cannabis use reveals new risk loci, genetic overlap with psychiatric traits, and a causal influence of schizophrenia. Nat Neurosci. 2018 Sep;21(9):1161–70.

2. Nikpay M, Goel A, Won HH, Hall LM, Willenborg C, Kanoni S, Saleheen D, Kyriakou T, Nelson CP, Hopewell JC, Webb TR, Zeng L, Dehghan A, Alver M, Armasu SM, Auro K, Bjonnes A, Chasman DI, Chen S, Ford I, Franceschini N, Gieger C, Grace C, Gustafsson S, Huang J, Hwang SJ, Kim YK, Kleber ME, Lau KW, Lu X, Lu Y, Lyytikäinen LP, Mihailov E, Morrison AC, Pervjakova N, Qu L, Rose LM, Salfati E, Saxena R, Scholz M, Smith AV, Tikkanen E, Uitterlinden A, Yang X, Zhang W, Zhao W, de Andrade M, de Vries PS, van Zuydam NR, Anand SS, Bertram L, Beutner F, Dedoussis G, Frossard P, Gauguier D, Goodall AH, Gottesman O, Haber M, Han BG, Huang J, Jalilzadeh S, Kessler T, König IR, Lannfelt L, Lieb W, Lind L, Lindgren CM, Lokki ML, Magnusson PK, Mallick NH, Mehra N, Meitinger T, Memon F ur R, Morris AP, Nieminen MS, Pedersen NL, Peters A, Rallidis LS, Rasheed A, Samuel M, Shah SH, Sinisalo J, Stirrups KE, Trompet S, Wang L, Zaman KS, Ardissino D, Boerwinkle E, Borecki IB, Bottinger EP, Buring JE, Chambers JC, Collins R, Cupples LA, Danesh J, Demuth I, Elosua R, Epstein SE, Esko T, Feitosa MF, Franco OH, Franzosi MG, Granger CB, Gu D, Gudnason V, Hall AS, Hamsten A, Harris TB, Hazen SL, Hengstenberg C, Hofman A, Ingelsson E, Iribarren C, Jukema JW, Karhunen PJ, Kim BJ, Kooner JS, Kullo IJ, Lehtimäki T, Loos RJF, Melander O, Metspalu A, März W, Palmer CN, Perola M, Quertermous T, Rader DJ, Ridker PM, Ripatti S, Roberts R, Salomaa V, Sanghera DK, Schwartz SM, Seedorf U, Stewart AF, Stott DJ, Thiery J, Zalloua PA, O’Donnell CJ, Reilly MP, Assimes TL, Thompson JR, Erdmann J, Clarke R, Watkins H, Kathiresan S, McPherson R, Deloukas P, Schunkert H, Samani NJ, Farrall M, the CARDIoGRAMplusC4D Consortium. A comprehensive 1000 Genomes–based genome-wide association meta-analysis of coronary artery disease. Nat Genet. 2015 Oct;47(10):1121–30.
